# Supplementary material for: A telomere-targeting drug depletes cancer initiating cells and promotes anti-tumor immunity in small cell lung cancer
Source: Nat Commun. 2024 Jan 22;15:672. doi: 10.1038/s41467-024-44861-8 (PMC10803750; doi:10.1038/s41467-024-44861-8)
Supplement: Supplementary file 1 — Supplementary Information [file 41467_2024_44861_MOESM1_ESM.pdf]

Figure S1

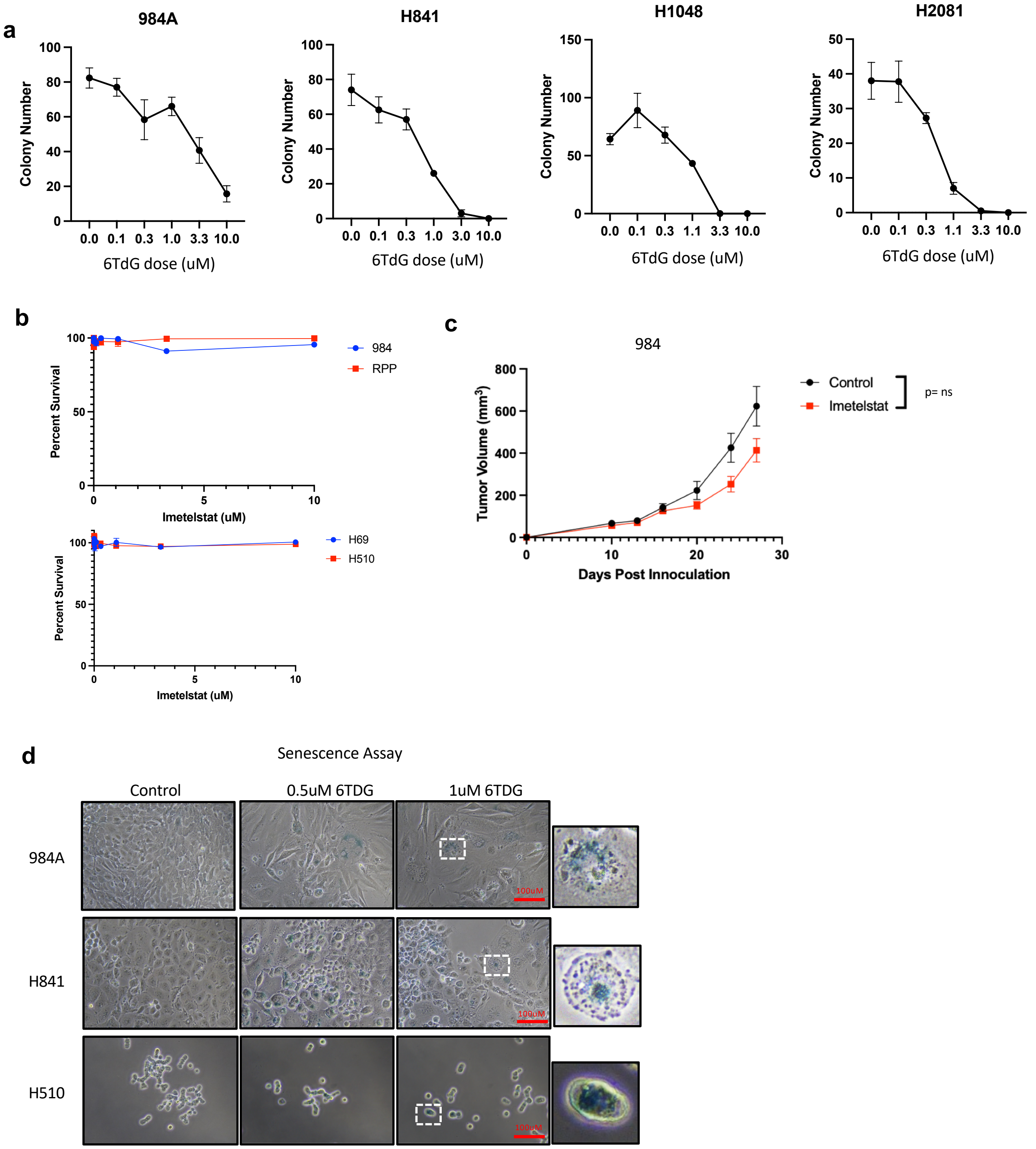
a
984A
H841
H1048
H2081
Colony Number
6TdG dose (uM)
0.0 0.1 0.3 1.0 3.3 10.0
0.0 0.1 0.3 1.0 3.0 10.0
0.0 0.1 0.3 1.1 3.3 10.0
b
Percent Survival
Imetelstat (uM)
984
RPP
H69
H510
c
984
Tumor Volume (mm3)
Days Post Innoculation
Control
Imetelstat
p= ns
d
Senescence Assay
Control
0.5uM 6TDG
1uM 6TDG
984A
H841
H510
100uM

Figure S1: 6TdG or imetelstat efficacy on SCLCs

a Colony formation assays were performed for 984A, H841, H2081 and H1048 cells treated with increasing concentrations of 6TdG. The number of colonies were quantified on the respective graphs. Replicates for for each condition 984A (n=3), H841 (n=2), H1048 (n=4), H2081 (n=4) . Error bars represent mean+ standard error of the mean (SEM). **b** IC50 curves of mouse SCLC cell lines 984 and RPP or human SCLC cell lines H69 and H510 treated with vehicle or increasing doses of Imetelstat. n=3 for each group. Error bars represent SEM. **c** Tumor growth curve of 984 cells subcutaneously implanted into WT mice and treated with vehicle (n=10) or 30mg/kg imetelstat (n=6) twice a week. Error bars represent SEM. Final tumor volumes were compared by an unpaired T test. **d** Representative images of vehicle, 0.5uM, or 1uM 6TdG treated 984A, H841, and H510 cells for senescence assay quantification. Senescent cells are shown in blue. N=5 replicates per condition. Source data are provided as a Source Data file.

Figure S2

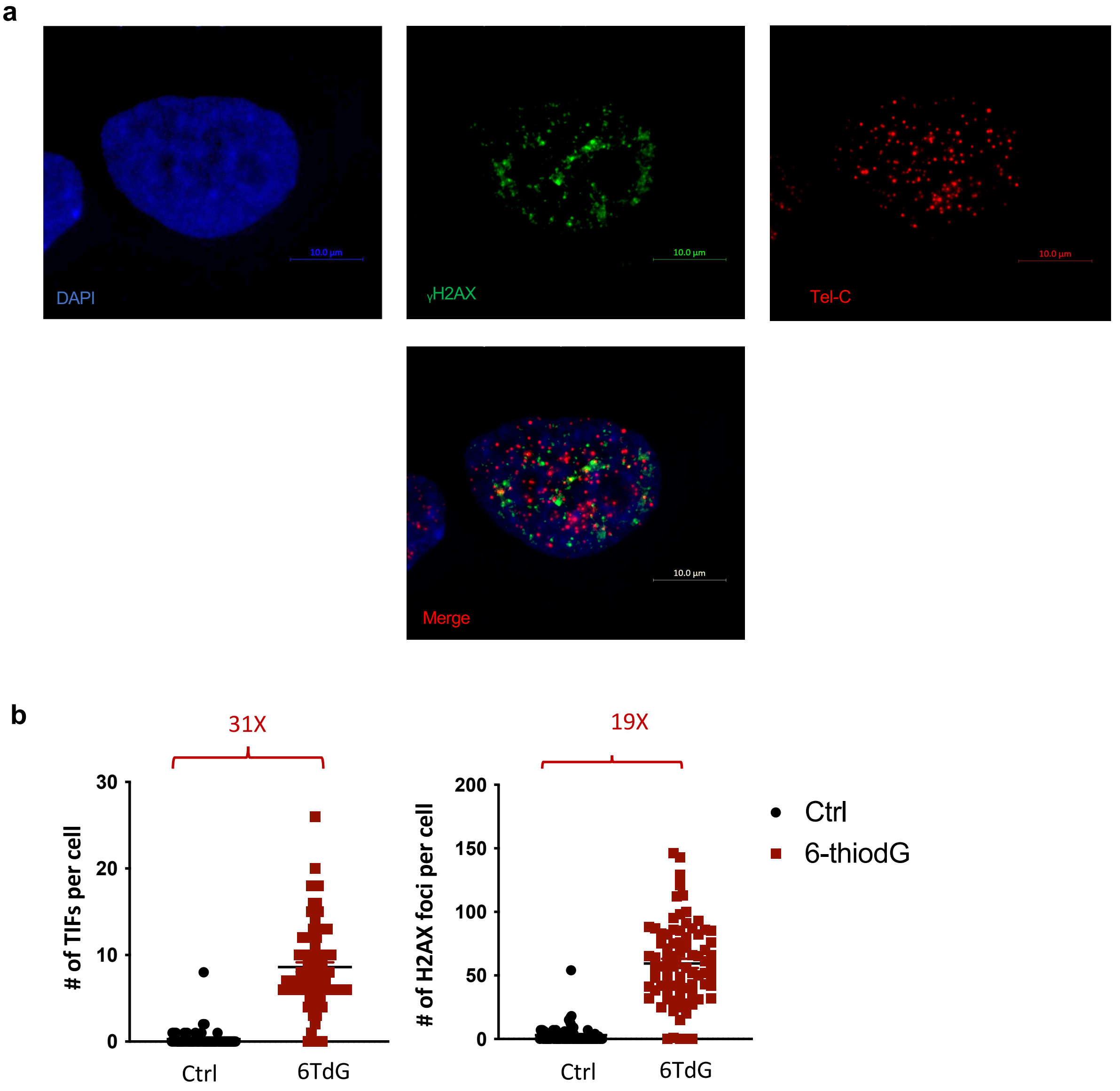
a
DAPI
10.0 µm
γH2AX
10.0 µm
Tel-C
10.0 µm
Merge
10.0 µm
b
31X
# of TIFs per cell
30
20
10
0
Ctrl
6TdG
19X
# of H2AX foci per cell
200
150
100
50
0
Ctrl
6TdG
Ctrl
6-thiodG

Figure S2: Analysis of double stranded DNA breaks

a Representative TIF images of control and 6TdG (5uM) treated H841 cells 48 hours after treatment. Nuclei were stained blue with DAPI, gH2AX was stained green and telomere probe (Tel-c) red. Co-localization of gH2AX and Tel-c foci are shown with yellow arrows. **b** Quantification of TIFs and gH2AX foci in control or 6TdG treated H841 cells. TIF quantification control sample has a minimum of 0, median of 0, and maximum of 8. 6TdG treated sample has a minimum of 0, median of 8, and maximum of 26. Mean for control is 0.2750 and for 6TdG treated 8.613. For gH2AX foci control has a minimum of 0, median of 0, and maximum of 54. For 6TdG treated has a minimum of 0, median of 57, and maximum of 146. The means were 3.125 and 59.44. n=80 for each group. Error bars represent mean+ standard deviation (SD). Source data are provided as a Source Data file.

Figure S3

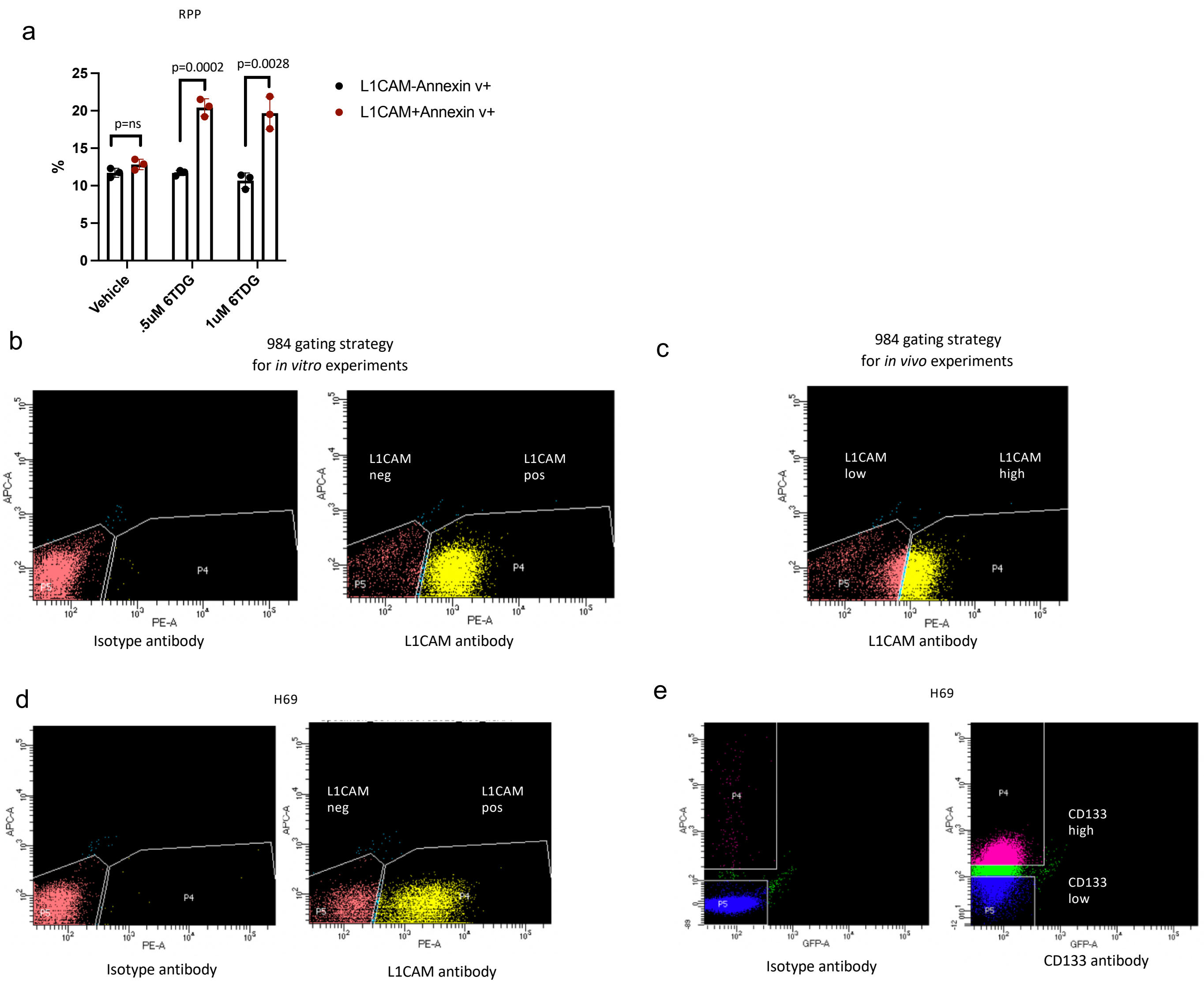

a
RPP
p=ns
p=0.0002
p=0.0028
%
25
20
15
10
5
0
Vehicle
.5uM 6TDG
1uM 6TDG
L1CAM-Annexin v+
L1CAM+Annexin v+
b
984 gating strategy
for *in vitro* experiments
APC-A
PE-A
P4
P5
L1CAM
neg
L1CAM
pos
Isotype antibody
L1CAM antibody
c
984 gating strategy
for *in vivo* experiments
L1CAM
low
L1CAM
high
L1CAM antibody
d
H69
Isotype antibody
L1CAM antibody
e
H69
GFP-A
CD133
high
CD133
low
Isotype antibody
CD133 antibody

Figure S3: Sensitivity of L1CAM positive cells to 6TdG

a Flow cytometry analysis of mouse RPP cell line treated with vehicle, 0.5uM, or 1uM 6TdG. After 48hr cells were harvested for flow cytometry and first gated on L1CAM positive and negative populations. Annexin V staining in these populations were quantified. All markers were normalized to isotype controls. Error bars represent mean+SEM. Values were compared by multiple unpaired T tests, n=3 replicates per condition. **b** Fluorescence-activated cell sorting (FACS) gating strategy used for experiments with 984 L1CAM negative and positive populations for *in vitro* experiments. **c** FACS gating strategy used for experiments with 984 L1CAM high and low populations for *in vivo* experiments **d** FACS gating strategy used for experiments with H69 L1CAM negative and positive populations for *in vitro* experiments. **e** FACS gating strategy used for experiments with H69 CD133 high and low populations for *in vitro* experiments. Source data are provided as a Source Data file.

Figure S4

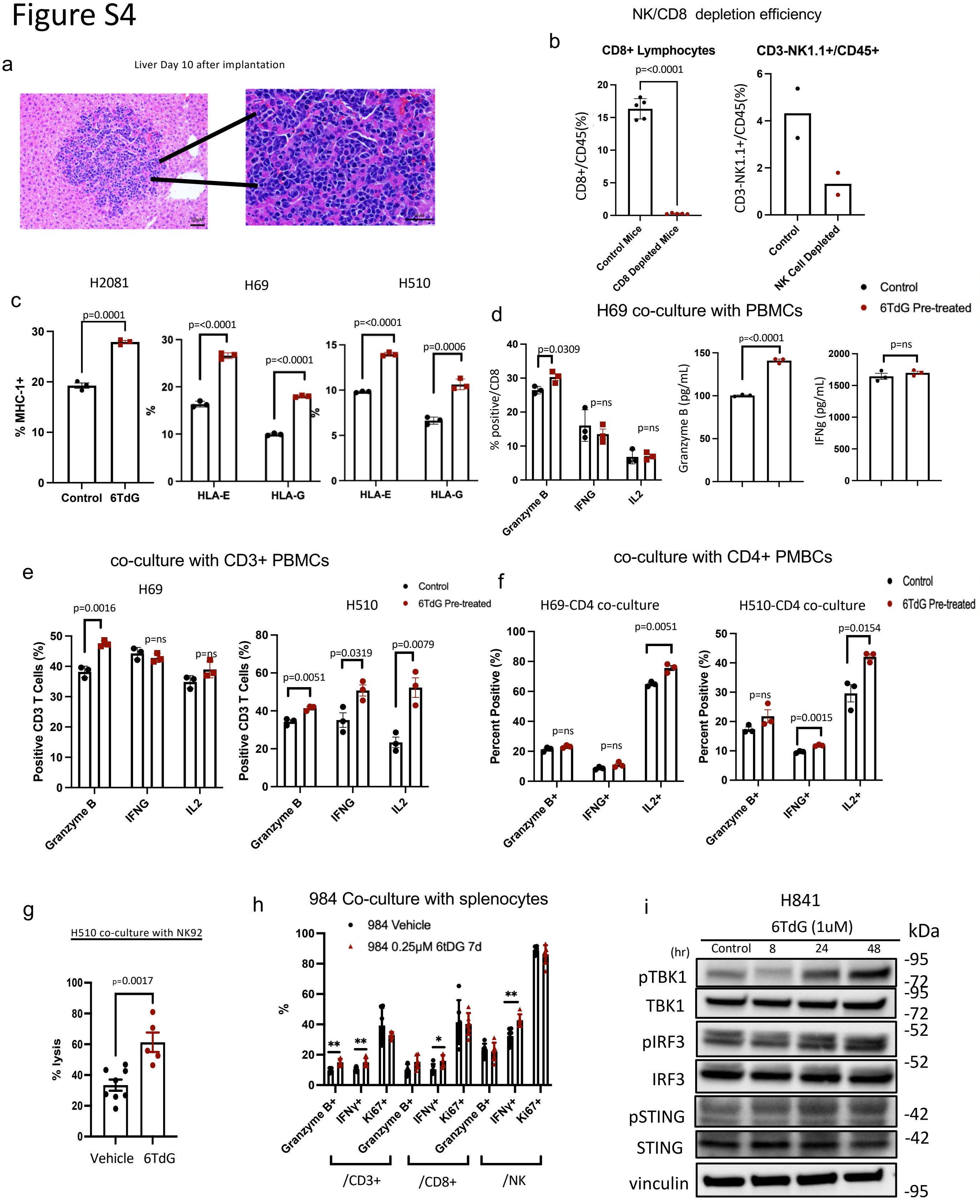
a
Liver Day 10 after implantation
b
NK/CD8 depletion efficiency
CD8+ Lymphocytes
CD3-NK1.1+/CD45+
p=<0.0001
CD8+/CD45(%)
CD3-NK1.1+/CD45(%)
Control Mice
CD8 Depleted Mice
Control
NK Cell Depleted
c
H2081
H69
H510
% MHC-1+
Control
6TdG
HLA-E
HLA-G
p=0.0001
p=<0.0001
p=0.0006
d
H69 co-culture with PBMCs
Control
6TdG Pre-treated
% positive/CD8
Granzyme B (pg/mL)
IFNg (pg/mL)
Granzyme B
IFNG
IL2
p=0.0309
p=ns
e
co-culture with CD3+ PBMCs
H69
H510
Positive CD3 T Cells (%)
p=0.0016
p=0.0051
p=0.0319
p=0.0079
f
co-culture with CD4+ PMBCs
H69-CD4 co-culture
H510-CD4 co-culture
Percent Positive (%)
Granzyme B+
IFNG+
IL2+
p=0.0051
p=0.0015
p=0.0154
g
H510 co-culture with NK92
% lysis
p=0.0017
Vehicle
6TdG
h
984 Co-culture with splenocytes
984 Vehicle
984 0.25μM 6tDG 7d
%
Granzyme B+
IFNγ+
Ki67+
/CD3+
/CD8+
/NK
i
H841
6TdG (1uM)
kDa
(hr)
Control
8
24
48
pTBK1
TBK1
pIRF3
IRF3
pSTING
STING
vinculin
-95
-72
-52
-42

Figure S4: Impact of 6TdG on tumor cells and lymphocytes in vitro

a Representative hematoxylin and eosin (H&E) images of liver tissue from WT mice injected with 984 cells and euthanatized 10 days after implantation, image is representative of n=5 mice. **b** NK or CD8T cell depletion efficiency in the blood antibody treated mice, n=3 for CD8 and 2 for NK depletion experiment. For CD8 depletion experiment graph error bars represent mean+SEM. **c** Flow cytometry analysis of human H2081 for MHC-1 and H69 or H510 for HLA-E/G cells treated with vehicle or 6TdG. N=3 for each group. Error bars represent mean+SEM. Statistical analysis was completed by multiple unpaired T tests. **d** Flow cytometry analysis on human PBMCs (gated on CD8+) co-cultured with vehicle or 6TdG pre-treated H69 cells (left) and granzyme B and IFNg in the co-culture media determined by ELISA (right). N=3 for each group. Error bars represent mean+SEM. Statistical analysis was completed by multiple unpaired T tests. **e** Flow cytometry analysis for IFNg, Granzyme-b and IL-2 of human CD3+ PBMCs co-cultured with vehicle or 6TdG pre-treated H69 or H510 cells. n=3 for each group. Error bars represent mean+SEM. Statistical analysis was completed by multiple unpaired T tests. **f** Flow cytometry analysis for IFNg, and IL2 of human CD4+ PBMCs co-cultured with vehicle or 6TdG pre-treated H69 or H510 cells. N=3 for each group. Error bars represent mean+SEM. Statistical analysis was completed by multiple unpaired T tests. **g** Vehicle or 6TdG pre-treated H510 cells were co-cultured with human NK-92 cell line. % of lysed of H510 is shown in vehicle (n=8) or 6TdG treated (p=5) cells. Error bars represent mean+SEM and statistical analysis completed by unpaired T test. **h**. Expression of Granzyme-B, IFNg and Ki67 was analyzed in CD3, CD8, and NK cell populations in splenocytes from wt mice co-cultured with vehicle or 6TdG pre-treated 984 cells. Error bars represent mean+SD. N=3 for each group. Out of CD3+ granzyme B+ (p=0.00083) and IFNg (p=0.0035). Out of CD8+ IFNg (p=0.0266). Out of NK IFNg (p=0.0032). Statistical analysis completed by multiple unpaired T tests. **i** Western blot analysis of TBK1, pTBK1, IRF3, pIRF3, STING, or pSTING, and vinculin in H841 cells treated with vehicle or 6TdG (1uM) for 0, 8, 24, or 48 hours. Experiment was repeated twice. Source data are provided as a Source Data file.

Figure S5

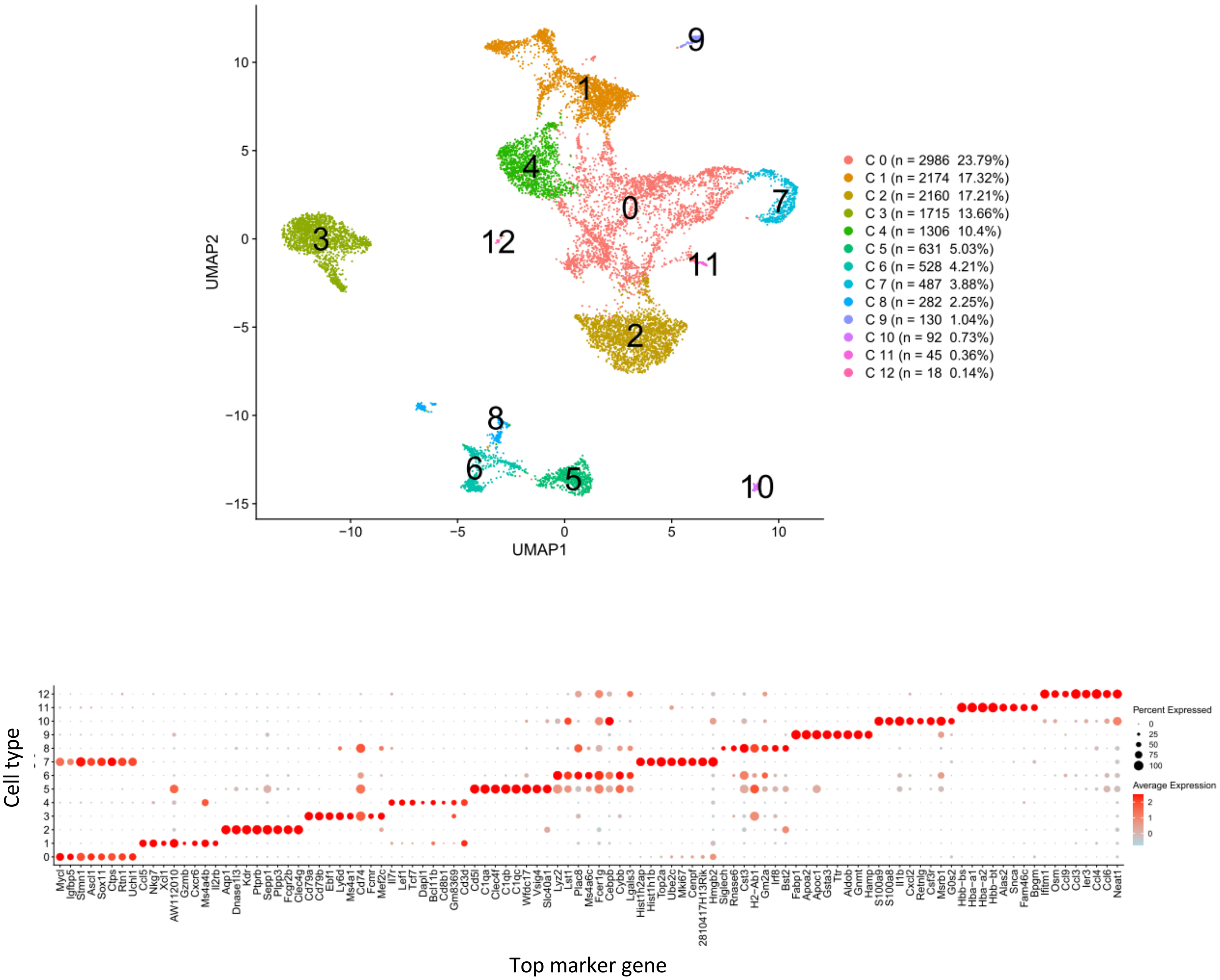
UMAP1
UMAP2
0
1
2
3
4
5
6
7
8
9
10
11
12
C 0 (n = 2986 23.79%)
C 1 (n = 2174 17.32%)
C 2 (n = 2160 17.21%)
C 3 (n = 1715 13.66%)
C 4 (n = 1306 10.4%)
C 5 (n = 631 5.03%)
C 6 (n = 528 4.21%)
C 7 (n = 487 3.88%)
C 8 (n = 282 2.25%)
C 9 (n = 130 1.04%)
C 10 (n = 92 0.73%)
C 11 (n = 45 0.36%)
C 12 (n = 18 0.14%)
Cell type
Top marker gene
Percent Expressed
0
25
50
75
100
Average Expression
Mycl Igfbp5 Stmn1 Ascl1 Sox11 Ctps Rtn1 Uchl1 Ccl5 Nkg7 Xcl1 AW112010 Gzmb Cxcr6 Ms4a4b Il2rb Aqp1 Dnase1l3 Kdr Ptprb Sepp1 Plpp3 Fcgr2b Clec4g Cd79a Cd79b Ebf1 Ly6d Ms4a1 Cd74 Fcmr Mef2c Il7r Lef1 Tcf7 Dapl1 Bcl11b Cd8b1 Gm8369 Cd3d Cd5l C1qa Clec4f C1qb C1qc Wfdc17 Vsig4 Slc40a1 Lyz2 Lst1 Plac8 Ms4a6c Fcer1g Cebpb Cybb Lgals3 Hist1h2ap Hist1h1b Top2a Ube2c Mki67 Cenpf 2810417H13Rik Hmgb2 Siglech Rnase6 Cst3 H2-Ab1 Gm2a Irf8 Bst2 Fabp1 Apoa2 Apoc1 Gsta3 Ttr Aldob Gnmt Hamp S100a9 S100a8 Il1b Cxcl2 Retnlg Csf3r Msrb1 G0s2 Hbb-bs Hba-a1 Hba-a2 Hbb-bt Alas2 Snca Fam46c Bpgm Ifitm1 Osm Ccl9 Ccl3 Ier3 Ccl4 Ccl6 Neat1

Figure S5: Cell type specific markers identified by single cell sequencing

Genes associated with major cell clusters identified from the single cell sequencing of the metastatic liver tumor tissues from vehicle or 6TdG treated mice are shown. Reads from the cells of vehicle or 6TdG treated mice were integrated for this clustering. Source data are provided as a Source Data file.

Figure S6

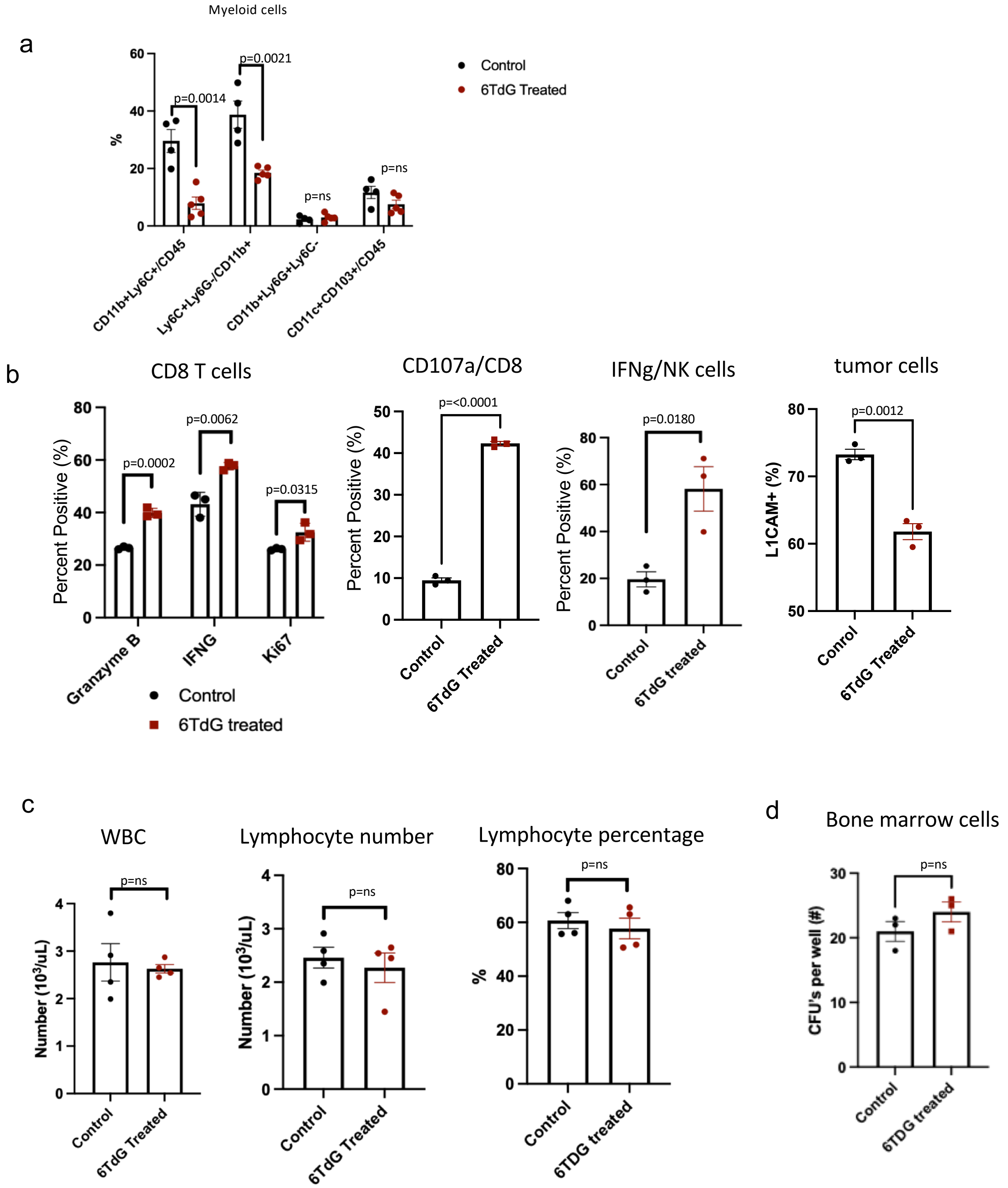

a
Myeloid cells
Control
6TdG Treated
%
p=0.0014
p=0.0021
p=ns
p=ns
CD11b+Ly6C+/CD45
Ly6C+Ly6G-/CD11b+
CD11b+Ly6G+Ly6C-
CD11c+CD103+/CD45
b
CD8 T cells
Percent Positive (%)
p=0.0002
p=0.0062
p=0.0315
Granzyme B
IFNG
Ki67
Control
6TdG treated
CD107a/CD8
p=<0.0001
Control
6TdG Treated
IFNg/NK cells
p=0.0180
Control
6TdG treated
tumor cells
L1CAM+ (%)
p=0.0012
Conrol
6TdG Treated
c
WBC
Number (10³/uL)
p=ns
Control
6TdG Treated
Lymphocyte number
p=ns
Lymphocyte percentage
%
p=ns
Control
6TDG treated
d
Bone marrow cells
CFU's per well (#)
p=ns
Control
6TDG treated

Figure S6: 6TdG induced changes in the tumor microenvironment

a Flow cytometry analysis of myeloid cell types in control and treated tumor bearing livers in WT mice IV injected with 984 cells. % positive cells were analyzed on graph. Error bars represent mean+SEM. N=4 for control and N=5 for 6TdG treated**. b** WT mice were injected subcutaneously with 984 mouse SCLC. Short term treatment with 6TdG or vehicle was performed. Mice were dosed with 3 consecutive doses as in figure 3 and sacked 5 days after the last dose for flow cytometry analysis. T cell populations and NK cell populations were analyzed by flow cytometry in control versus treated groups. The percentage of L1CAM positive tumor cells in vehicle or 6TdG treated groups were quantified. N=3 for each group. Error bars represents mean+SEM. Multiple unpaired T tests were used for statistical analysis**. c** WBC count, lymphocyte number, and lymphocyte percentage were analyzed from the blood of long-term WT mice treated with control or 6TdG. n=4 for each group. Error bars represents mean+SEM. Multiple unpaired T tests were used for statistical analysis. **d** Bone marrow cells were collected from WT mice treated with vehicle (n=3) or 6TdG (n=3) and grown in Methocult cell differentiation media. After 10 days the number of colony forming units per well were counted and graphed. Error bars represents SEM. Source data are provided as a Source Data file.

Figure S7

a

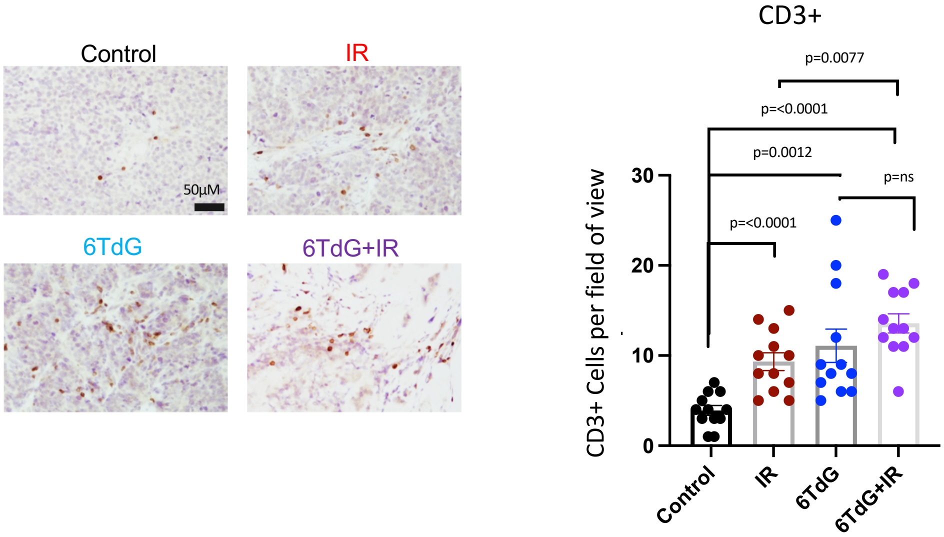
Control
IR
50μM
6TdG
6TdG+IR
CD3+
p=0.0077
p=<0.0001
p=0.0012
p=ns
p=<0.0001
CD3+ Cells per field of view
30
20
10
0
Control
IR
6TdG
6TdG+IR

Figure S7: T cell presence in 6TdG, IR or combination treated mice

Immunohistochemistry for CD3 on subcutaneous tumor tissue of WT mice with 984 flank tumors treated with vehicle, 6TdG, IR or combination of 6TdG and IR. Representative images are shown on the left and quantification of CD3+ cells per microscopic field is shown on the right. N=12 for each group. Error bars represent mean+SEM. Unpaired T tests were used for statistical analysis. Source data are provided as a Source Data file.

Figure S8

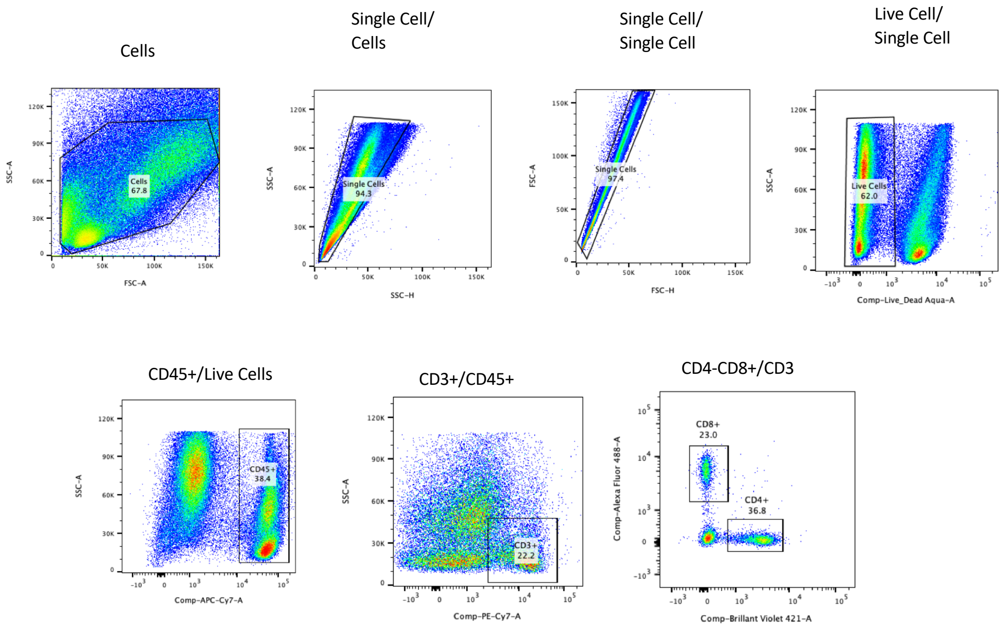
Cells
Cells
67.8
SSC-A
FSC-A
Single Cell/
Cells
Single Cells
94.3
SSC-A
SSC-H
Single Cell/
Single Cell
Single Cells
97.4
FSC-A
FSC-H
Live Cell/
Single Cell
Live Cells
62.0
SSC-A
Comp-Live_Dead Aqua-A
CD45+/Live Cells
CD45+
38.4
SSC-A
Comp-APC-Cy7-A
CD3+/CD45+
CD3+
22.2
SSC-A
Comp-PE-Cy7-A
CD4-CD8+/CD3
CD8+
23.0
CD4+
36.8
Comp-Alexa Fluor 488-A
Comp-Brillant Violet 421-A

Figure S8: Gating strategy

Example mouse tumor gating using FlowJo, related to methods and main figure 6. Source data are provided as a Source Data file.

Tale S1. List of Antibodies Used

Company	Cat #	Antibody	Dilution	Incubation Time	Validation	
**Flow Cytometry**						
Biolegend	304006	Alexa Fluor® 488 anti-human CD45	1/100	20 min on ice	Each lot of this antibody is quality control tested by immunofluorescent staining with flow cytometric analysis by the manufacturer. See the manufacturer page for validation data and references:	https://www.biolegend.com/en-us/products/fitc-anti-human-cd45-antibody-707
Biolegend	320912	Alexa Fluor® 488 anti-human MICA/MICB	1/100	20 min on ice	Each lot of this antibody is quality control tested by immunofluorescent staining with flow cytometric analysis by the manufacturer. See the manufacturer page for validation data and references:	https://www.biolegend.com/en-us/products/alexa-fluor-488-anti-human-mica-micb-antibody-3067
Biolegend	100423	Alexa Fluor® 488 anti-mouse CD4	1/100	20 min on ice	Each lot of this antibody is quality control tested by immunofluorescent staining with flow cytometric analysis by the manufacturer. See the manufacturer page for validation data and references:	https://www.biolegend.com/en-us/products/alexa-fluor-488-anti-mouse-cd4-antibody-2695
Biolegend	103122	Alexa Fluor® 488 anti-mouse CD45	1/100	20 min on ice	Each lot of this antibody is quality control tested by immunofluorescent staining with flow cytometric analysis by the manufacturer. See the manufacturer page for validation data and references:	https://www.biolegend.com/en-us/products/alexa-fluor-488-anti-mouse-cd45-antibody-3100
Biolegend	108913	Alexa Fluor® 488 anti-mouse CD49b (pan-NK cells)	1/100	20 min on ice	Each lot of this antibody is quality control tested by immunofluorescent staining with flow cytometric analysis by the manufacturer. See the manufacturer page for validation data and references:	https://www.biolegend.com/en-us/products/alexa-fluor-488-anti-mouse-cd49b-pan-nk-cells-antibody-2709
Biolegend	100723	Alexa Fluor® 488 anti-mouse CD8a	1/100	20 min on ice	Each lot of this antibody is quality control tested by immunofluorescent staining with flow cytometric analysis by the manufacturer. See the manufacturer page for validation data and references:	https://www.biolegend.com/en-us/products/alexa-fluor-488-anti-mouse-cd8a-antibody-2698
Biolegend	100516	APC anti-mouse CD4	1/100	20 min on ice	Each lot of this antibody is quality control tested by immunofluorescent staining with flow cytometric analysis by the manufacturer. See the manufacturer page for validation data and references:	https://www.biolegend.com/en-us/products/apc-anti-mouse-cd4-antibody-477
Biolegend	503810	APC anti-mouse IL-2	1/100	20 min on ice	Each lot of this antibody is quality control tested by immunofluorescent staining with flow cytometric analysis by the manufacturer. See the manufacturer page for validation data and references:	https://www.biolegend.com/en-us/products/apc-anti-mouse-il-2-antibody-950
Biolegend	400612	APC Rat IgG2b, κ Isotype Ctrl	1/100	20 min on ice	Each lot of this antibody is quality control tested by immunofluorescent staining with flow cytometric analysis as negative control by the manufacturer. See the manufacturer page for validation data and references:	https://www.biolegend.com/en-us/products/apc-rat-igg2b-kappa-isotype-ctrl-1851
Biolegend	103116	APC/Cy7 anti-mouse CD45	1/100	20 min on ice	Each lot of this antibody is quality control tested by immunofluorescent staining with flow cytometric analysis by the manufacturer. See the manufacturer page for validation data and references:	https://www.biolegend.com/en-us/products/apc-cyanine7-anti-mouse-cd45-antibody-2530
Biolegend	100714	APC/Cy7 anti-mouse CD8a	1/100	20 min on ice	Each lot of this antibody is quality control tested by immunofluorescent staining with flow cytometric analysis by the manufacturer. See the manufacturer page for validation data and references:	https://www.biolegend.com/en-us/products/apc-cyanine7-anti-mouse-cd8a-antibody-2269
Biolegend	115538	Brilliant Violet 421™ anti-mouse CD19	1/100	20 min on ice	Each lot of this antibody is quality control tested by immunofluorescent staining with flow cytometric analysis by the manufacturer. See the manufacturer page for validation data and references:	https://www.biolegend.com/en-us/products/brilliant-violet-421-anti-mouse-cd19-antibody-7160
Biolegend	652411	Brilliant Violet 421™ anti-mouse Ki-67	1/100	20 min on ice	Each lot of this antibody is quality control tested by immunofluorescent staining with flow cytometric analysis by the manufacturer. See the manufacturer page for validation data and references:	https://www.biolegend.com/en-us/products/brilliant-violet-421-anti-mouse-ki-67-antibody-8982
Biolegend	118216	PE/Cy7 anti-mouse CD326 (Ep-CAM)	1/100	20 min on ice	Each lot of this antibody is quality control tested by immunofluorescent staining with flow cytometric analysis by the manufacturer. See the manufacturer page for validation data and references:	https://www.biolegend.com/en-us/products/pe-cyanine7-anti-mouse-cd326-ep-cam-antibody-5303
Biolegend	100320	PE/Cy7 anti-mouse CD3ε	1/100	20 min on ice	Each lot of this antibody is quality control tested by immunofluorescent staining with flow cytometric analysis by the manufacturer. See the manufacturer page for validation data and references:	https://www.biolegend.com/en-us/products/pe-cyanine7-anti-mouse-cd3epsilon-antibody-1899
Biolegend	324214	PerCP/Cy5.5 anti-human CD326 (Ep-CAM)	1/100	20 min on ice	Each lot of this antibody is quality control tested by immunofluorescent staining with flow cytometric analysis by the manufacturer. See the manufacturer page for validation data and references:	https://www.biolegend.com/en-us/products/percp-cyanine5-5-anti-human-cd326-epcam-antibody-4252
Biolegend	137610	PerCP/Cy5.5 anti-mouse CD335 (NKp46)	1/100	20 min on ice	Each lot of this antibody is quality control tested by immunofluorescent staining with flow cytometric analysis by the manufacturer. See the manufacturer page for validation data and references:	https://www.biolegend.com/en-us/products/percp-cyanine5-5-anti-mouse-cd335-nkp46-antibody-6724
Biolegend	400233	Alexa Fluor® 488 Mouse IgG2a, κ Isotype Ctrl Antibody	1/100	20 min on ice	Each lot of this antibody is quality control tested by immunofluorescent staining with flow cytometric analysis by the manufacturer. See the manufacturer page for validation data and references:	https://www.biolegend.com/en-us/products/alexa-fluor-488-mouse-igg2a-kappa-isotype-ctrl-2690
Biolegend	400636	PE Rat IgG2b, κ Isotype Ctrl Antibody	1/100	20 min on ice	Each lot of this antibody is quality control tested by immunofluorescent staining with flow cytometric analysis by the manufacturer. See the manufacturer page for validation data and references:	https://www.biolegend.com/en-us/products/pe-rat-igg2b-kappa-isotype-ctrl-1856
Biolegend	400220	APC Mouse IgG2a, κ Isotype Ctrl Antibody	1/100	20 min on ice	Each lot of this antibody is quality control tested by immunofluorescent staining with flow cytometric analysis by the manufacturer. See the manufacturer page for validation data and references:	https://www.biolegend.com/en-us/products/apc-mouse-igg2a-kappa-isotype-ctrl-1397
Biolegend	311410	APC anti-human HLA-A,B,C Antibody	1/100	20 min on ice	Each lot of this antibody is quality control tested by immunofluorescent staining with flow cytometric analysis by the manufacturer. See the manufacturer page for validation data and references:	https://www.biolegend.com/en-us/products/apc-anti-human-hla-a-b-c-antibody-1870
Biolegend	137607	APC anti-mouse CD335 (NKp46) Antibody	1/100	20 min on ice	Each lot of this antibody is quality control tested by immunofluorescent staining with flow cytometric analysis by the manufacturer. See the manufacturer page for validation data and references:	https://www.biolegend.com/en-us/products/apc-anti-mouse-cd335-nkp46-antibody-6676
Biolegend	114607	PE anti-mouse H-2K b/H-2D b Antibody	1/100	20 min on ice	Each lot of this antibody is quality control tested by immunofluorescent staining with flow cytometric analysis by the manufacturer. See the manufacturer page for validation data and references:	https://www.biolegend.com/en-us/products/pe-anti-mouse-h-2k-b-h-2d-b-antibody-1686
Biolegend	400212	PE Mouse IgG2a, κ Isotype Ctrl Antibody	1/100	20 min on ice	Each lot of this antibody is quality control tested by immunofluorescent staining with flow cytometric analysis as negative control by the manufacturer. See the manufacturer page for validation data and references:	https://www.biolegend.com/en-us/products/pe-mouse-igg2a-kappa-isotype-ctrl-1401
Biolegend	400341	Brilliant Violet 421™ Mouse IgG2b, κ Isotype Ctrl Antibody	1/100	20 min on ice	Each lot of this antibody is quality control tested by immunofluorescent staining with flow cytometric analysis as negative control by the manufacturer. See the manufacturer page for validation data and references:	https://www.biolegend.com/en-us/products/brilliant-violet-421-mouse-igg2b-kappa-isotype-ctrl-7195
Biolegend	133203	PE anti-mouse RAE-1δ Antibody	1/100	20 min on ice	Each lot of this antibody is quality control tested by immunofluorescent staining with flow cytometric analysis by the manufacturer. See the manufacturer page for validation data and references:	https://www.biolegend.com/en-us/products/pe-anti-mouse-rae-1delta-antibody-5768
Biolegend	400111	PE Mouse IgG1, κ Isotype Ctrl Antibody	1/100	20 min on ice	Each lot of this antibody is quality control tested by immunofluorescent staining with flow cytometric analysis as negative control by the manufacturer. See the manufacturer page for validation data and references:	https://www.biolegend.com/en-us/products/pe-mouse-igg1-kappa-isotype-ctrl-1408
Biolegend	400319	APC Mouse IgG2b, κ Isotype Ctrl Antibody	1/100	20 min on ice	Each lot of this antibody is quality control tested by immunofluorescent staining with flow cytometric analysis as negative control by the manufacturer. See the manufacturer page for validation data and references:	https://www.biolegend.com/en-us/products/apc-mouse-igg2b-kappa-isotype-ctrl-1410
Biolegend	410711	APC anti-human IgG Fc Antibody	1/100	20 min on ice	Each lot of this antibody is quality control tested by immunofluorescent staining with flow cytometric analysis by the manufacturer. See the manufacturer page for validation data and references:	https://www.biolegend.com/en-us/products/apc-anti-human-igg-fc-11935
Biolegend	400511	APC Rat IgG2a, κ Isotype Ctrl Antibody	1/100	20 min on ice	Each lot of this antibody is quality control tested by immunofluorescent staining with flow cytometric analysis as negative control by the manufacturer. See the manufacturer page for validation data and references:	https://www.biolegend.com/en-us/products/apc-rat-igg2a-kappa-isotype-ctrl-1838
Biolegend	400119	APC Mouse IgG1, κ Isotype Ctrl Antibody	1/100	20 min on ice	Each lot of this antibody is quality control tested by immunofluorescent staining with flow cytometric analysis as negative control by the manufacturer. See the manufacturer page for validation data and references:	https://www.biolegend.com/en-us/products/apc-mouse-igg1-kappa-isotype-ctrl-1404
Biolegend	100733	PerCP/Cy5.5 anti-mouse CD8a Antibody	1/100	20 min on ice	Each lot of this antibody is quality control tested by immunofluorescent staining with flow cytometric analysis by the manufacturer. See the manufacturer page for validation data and references:	https://www.biolegend.com/en-us/products/percp-cyanine5-5-anti-mouse-cd8a-antibody-4255
Biolegend	407110	APC anti-mouse IgG2a Antibody	1/100	20 min on ice	Each lot of this antibody is quality control tested by immunofluorescent staining with flow cytometric analysis by the manufacturer. See the manufacturer page for validation data and references:	https://www.biolegend.com/en-us/products/apc-anti-mouse-igg2a-8327
Biolegend	400411	APC Rat IgG1, κ Isotype Ctrl Antibody	1/100	20 min on ice	Each lot of this antibody is quality control tested by immunofluorescent staining with flow cytometric analysis as negative control by the manufacturer. See the manufacturer page for validation data and references:	https://www.biolegend.com/en-us/products/apc-rat-igg1-kappa-isotype-ctrl-1826
Biolegend	331913	Brilliant Violet 421™ anti-human CD335 (NKp46) Antibody	1/100	20 min on ice	Each lot of this antibody is quality control tested by immunofluorescent staining with flow cytometric analysis by the manufacturer. See the manufacturer page for validation data and references:	https://www.biolegend.com/en-us/products/brilliant-violet-421-anti-human-cd335-nkp46-antibody-7529
Biolegend	400157	Brilliant Violet 421™ Mouse IgG1, κ Isotype Ctrl Antibody	1/100	20 min on ice	Each lot of this antibody is quality control tested by immunofluorescent staining with flow cytometric analysis as negative control by the manufacturer. See the manufacturer page for validation data and references:	https://www.biolegend.com/en-us/products/brilliant-violet-421-mouse-igg1-kappa-isotype-ctrl-7194
Biolegend	400125	PE/Cy7 Mouse IgG1, κ Isotype Ctrl Antibody	1/100	20 min on ice	Each lot of this antibody is quality control tested by immunofluorescent staining with flow cytometric analysis as negative control by the manufacturer. See the manufacturer page for validation data and references:	https://www.biolegend.com/en-us/products/pe-cyanine7-mouse-igg1-kappa-isotype-ctrl-1926
Biolegend	100328	PerCP/Cy5.5 anti-mouse CD3ε Antibody	1/100	20 min on ice	Each lot of this antibody is quality control tested by immunofluorescent staining with flow cytometric analysis by the manufacturer. See the manufacturer page for validation data and references:	https://www.biolegend.com/en-us/products/percp-cyanine5-5-anti-mouse-cd3epsilon-antibody-4191
Biolegend	100321	Alexa Fluor® 488 anti-mouse CD3ε Antibody	1/100	20 min on ice	Each lot of this antibody is quality control tested by immunofluorescent staining with flow cytometric analysis by the manufacturer. See the manufacturer page for validation data and references:	https://www.biolegend.com/en-us/products/alexa-fluor-488-anti-mouse-cd3epsilon-antibody-2676

Biolegend	100512	PE anti-mouse CD4 Antibody	1/100	20 min on ice	Each lot of this antibody is quality control tested by immunofluorescent staining with flow cytometric analysis by the manufacturer. See the manufacturer page for validation data and references:	https://www.biolegend.com/en-us/products/pe-anti-mouse-cd4-antibody-482
Biolegend	121614	APC anti-mouse CD107a (LAMP-1) Antibody	1/100	20 min on ice	Each lot of this antibody is quality control tested by immunofluorescent staining with flow cytometric analysis by the manufacturer. See the manufacturer page for validation data and references:	https://www.biolegend.com/en-us/products/apc-anti-mouse-cd107a-lamp-1-antibody-6081
Biolegend	121626	PerCP/Cy5.5 anti-mouse CD107a (LAMP-1) Antibody	1/100	20 min on ice	Each lot of this antibody is quality control tested by immunofluorescent staining with flow cytometric analysis by the manufacturer. See the manufacturer page for validation data and references:	https://www.biolegend.com/en-us/products/percp-cyanine5-5-anti-mouse-cd107a-lamp-1-antibody-13079
Biolegend	400549	Brilliant Violet 421™ Rat IgG2a, κ Isotype Ctrl Antibody	1/100	20 min on ice	Each lot of this antibody is quality control tested by immunofluorescent staining with flow cytometric analysis as negative control by the manufacturer. See the manufacturer page for validation data and references:	https://www.biolegend.com/en-us/products/brilliant-violet-421-rat-igg2a-kappa-isotype-ctrl-7135
Biolegend	503808	PE anti-mouse IL-2 Antibody	1/100	20 min on ice	Each lot of this antibody is quality control tested by immunofluorescent staining with flow cytometric analysis by the manufacturer. See the manufacturer page for validation data and references:	https://www.biolegend.com/en-us/products/pe-anti-mouse-il-2-antibody-954
Biolegend	101320	TruStain fcX™ (anti-mouse CD16/32) Antibody	1/100	20 min on ice	Each lot of this antibody is quality control tested by immunofluorescent staining with flow cytometric analysis by the manufacturer. See the manufacturer page for validation data and references:	https://www.biolegend.com/en-us/products/trustain-fcx-anti-mouse-cd16-32-antibody-5683
Biolegend	100341	Brilliant Violet 421™ anti-mouse CD3ε Antibody	1/100	20 min on ice	Each lot of this antibody is quality control tested by immunofluorescent staining with flow cytometric analysis by the manufacturer. See the manufacturer page for validation data and references:	https://www.biolegend.com/en-us/products/brilliant-violet-421-anti-mouse-cd3epsilon-antibody-7132
Biolegend	400408	PE Rat IgG1, κ Isotype Ctrl Antibody	1/100	20 min on ice	Each lot of this antibody is quality control tested by immunofluorescent staining with flow cytometric analysis as negative control by the manufacturer. See the manufacturer page for validation data and references:	https://www.biolegend.com/en-us/products/pe-rat-igg1-kappa-isotype-ctrl-1830
Biolegend	400326	PE/Cy7 Mouse IgG2b, κ Isotype Ctrl Antibody	1/100	20 min on ice	Each lot of this antibody is quality control tested by immunofluorescent staining with flow cytometric analysis as negative control by the manufacturer. See the manufacturer page for validation data and references:	https://www.biolegend.com/en-us/products/pe-cyanine7-mouse-igg2b-kappa-isotype-ctrl-1928
Biolegend	100320	PE/Cy7 anti-mouse CD3ε Antibody	1/100	20 min on ice	Each lot of this antibody is quality control tested by immunofluorescent staining with flow cytometric analysis by the manufacturer. See the manufacturer page for validation data and references:	https://www.biolegend.com/en-us/products/pe-cyanine7-anti-mouse-cd3epsilon-antibody-1899
Biolegend	400508	PE Rat IgG2a, κ Isotype Ctrl Antibody	1/100	20 min on ice	Each lot of this antibody is quality control tested by immunofluorescent staining with flow cytometric analysis as negative control by the manufacturer. See the manufacturer page for validation data and references:	https://www.biolegend.com/en-us/products/pe-rat-igg2a-kappa-isotype-ctrl-1843
Biolegend	311406	PE anti-human HLA-A,B,C Antibody	1/100	20 min on ice	Each lot of this antibody is quality control tested by immunofluorescent staining with flow cytometric analysis by the manufacturer. See the manufacturer page for validation data and references:	https://www.biolegend.com/en-us/products/pe-anti-human-hla-a-b-c-antibody-1872
Biolegend	400214	PE Mouse IgG2a, κ Isotype Ctrl (FC) Antibody	1/100	20 min on ice	Each lot of this antibody is quality control tested by immunofluorescent staining with flow cytometric analysis as negative control by the manufacturer. See the manufacturer page for validation data and references:	https://www.biolegend.com/en-us/products/pe-mouse-igg2a-kappa-isotype-ctrl-fc-3043
Biolegend	114608	PE anti-mouse H-2Kb/H-2Db Antibody	1/100	20 min on ice	Each lot of this antibody is quality control tested by immunofluorescent staining with flow cytometric analysis by the manufacturer. See the manufacturer page for validation data and references:	https://www.biolegend.com/en-us/products/pe-anti-mouse-h-2k-b-h-2d-b-antibody-1686
Biolegend	503826	Brilliant Violet 421™ anti-mouse IL-2 Antibody	1/100	20 min on ice	Each lot of this antibody is quality control tested by immunofluorescent staining with flow cytometric analysis by the manufacturer. See the manufacturer page for validation data and references:	https://www.biolegend.com/en-us/products/brilliant-violet-421-anti-mouse-il-2-antibody-7202
Biolegend	506510	APC anti-human IFN-γ Antibody	1/100	20 min on ice	Each lot of this antibody is quality control tested by immunofluorescent staining with flow cytometric analysis by the manufacturer. See the manufacturer page for validation data and references:	https://www.biolegend.com/en-us/products/apc-anti-human-ifn-gamma-antibody-1533
Biolegend	300316	PE/Cy7 anti-human CD3 Antibody	1/100	20 min on ice	Each lot of this antibody is quality control tested by immunofluorescent staining with flow cytometric analysis by the manufacturer. See the manufacturer page for validation data and references:	https://www.biolegend.com/en-us/products/pe-cyanine7-anti-human-cd3-antibody-1913
Biolegend	372206	FITC anti-human/mouse Granzyme B Recombinant Antibody	1/100	20 min on ice	Each lot of this antibody is quality control tested by immunofluorescent staining with flow cytometric analysis by the manufacturer. See the manufacturer page for validation data and references:	https://www.biolegend.com/en-us/products/fitc-anti-human-mouse-granzyme-b-recombinant-antibody-14430
Biolegend	100734	PerCP/Cyanine5.5 anti-mouse CD8a Antibody	1/100	20 min on ice	Each lot of this antibody is quality control tested by immunofluorescent staining with flow cytometric analysis by the manufacturer. See the manufacturer page for validation data and references:	https://www.biolegend.com/en-us/products/percp-cyanine5-5-anti-mouse-cd8a-antibody-4255
Biolegend	400138	FITC Mouse IgG1, κ Isotype Ctrl (ICFC) Antibody	1/100	20 min on ice	Each lot of this antibody is quality control tested by immunofluorescent staining with flow cytometric analysis as negative control by the manufacturer. See the manufacturer page for validation data and references:	https://www.biolegend.com/en-us/products/fitc-mouse-igg1-kappa-isotype-ctrl-icfc-3031
Biolegend	108708	PE anti-mouse NK-1.1 Antibody	1/100	20 min on ice	Each lot of this antibody is quality control tested by immunofluorescent staining with flow cytometric analysis by the manufacturer. See the manufacturer page for validation data and references:	https://www.biolegend.com/en-us/products/pe-anti-mouse-nk-1-1-antibody-431
Biolegend	108913	Alexa Fluor® 488 anti-mouse CD49b (pan-NK cells) Antibody	1/100	20 min on ice	Each lot of this antibody is quality control tested by immunofluorescent staining with flow cytometric analysis by the manufacturer. See the manufacturer page for validation data and references:	https://www.biolegend.com/en-us/products/alexa-fluor-488-anti-mouse-cd49b-pan-nk-cells-antibody-2709
Biolegend	108910	APC anti-mouse CD49b (pan-NK cells) Antibody	1/100	20 min on ice	Each lot of this antibody is quality control tested by immunofluorescent staining with flow cytometric analysis by the manufacturer. See the manufacturer page for validation data and references:	https://www.biolegend.com/en-us/products/apc-anti-mouse-cd49b-pan-nk-cells-antibody-231
Biolegend	400522	PE/Cy7 Rat IgG2a, κ Isotype Ctrl Antibody	1/100	20 min on ice	Each lot of this antibody is quality control tested by immunofluorescent staining with flow cytometric analysis as negative control by the manufacturer. See the manufacturer page for validation data and references:	https://www.biolegend.com/en-us/products/pe-cyanine7-rat-igg2a-kappa-isotype-ctrl-1935
Biolegend	400617	PE/Cy7 Rat IgG2b, κ Isotype Ctrl Antibody	1/100	20 min on ice	Each lot of this antibody is quality control tested by immunofluorescent staining with flow cytometric analysis as negative control by the manufacturer. See the manufacturer page for validation data and references:	https://www.biolegend.com/en-us/products/pe-cyanine7-rat-igg2b-kappa-isotype-ctrl-1936
Biolegend	324221	PE/Cy7 anti-human CD326 (EpCAM) Antibody	1/100	20 min on ice	Each lot of this antibody is quality control tested by immunofluorescent staining with flow cytometric analysis by the manufacturer. See the manufacturer page for validation data and references:	https://www.biolegend.com/en-us/products/pe-cyanine7-anti-human-cd326-epcam-antibody-8107
Biolegend	304014	APC/Cyanine7 anti-human CD45 Antibody	1/100	20 min on ice	Each lot of this antibody is quality control tested by immunofluorescent staining with flow cytometric analysis by the manufacturer. See the manufacturer page for validation data and references:	https://www.biolegend.com/en-us/products/apc-cyanine7-anti-human-cd45-antibody-1914
Biolegend	320906	PE anti-human MICA/MICB Antibody	1/100	20 min on ice	Each lot of this antibody is quality control tested by immunofluorescent staining with flow cytometric analysis by the manufacturer. See the manufacturer page for validation data and references:	https://www.biolegend.com/en-us/products/pe-anti-human-mica-micb-antibody-3064
Biolegend	505808	PE anti-mouse IFN-γ Antibody	1/100	20 min on ice	Each lot of this antibody is quality control tested by immunofluorescent staining with flow cytometric analysis by the manufacturer. See the manufacturer page for validation data and references:	https://www.biolegend.com/en-us/products/pe-anti-mouse-ifn-gamma-antibody-997
Biolegend	103116	APC/Cyanine7 anti-mouse CD45 Antibody	1/100	20 min on ice	Each lot of this antibody is quality control tested by immunofluorescent staining with flow cytometric analysis by the manufacturer. See the manufacturer page for validation data and references:	https://www.biolegend.com/en-us/products/apc-cyanine7-anti-mouse-cd45-antibody-2530
R&D	666-DN	Human DNAM-1/CD226 Fc Chimera Recombinant Protein	1/100	20 min on ice	Activity is measured by its binding ability in a functional ELISA.When Recombinant Human DNAM-1 Fc Chimera (Catalog # 666-DN) is immobilized at 1 µg/mL, 100 µL/well, Biotinylated Recombinant Human CD155/PVR (Catalog # BT9174) binds with an ED50 of 3-18 ng/mL. See manufacturer's website for validation information:	https://www.rndsystems.com/products/recombinant-human-dnam-1-cd226-fc-chimera-protein-cf_666-dn#product-datasheets
R&D	4436-DN	Mouse DNAM-1/CD226 Fc Chimera Recombinant Protein	1/100	20 min on ice	Activity is measured by its binding ability in a functional ELISA.When Recombinant Mouse Nectin-2/ CD112 (Catalog # 3869-N2) is coated at 1 µg/mL, Recombinant Mouse DNAM-1/CD226 Fc Chimera (Catalog # 4436-DN) binds with a typical ED50 of 0.3-1.8 µg/mL.See manufacturer's website for validation information:	https://www.rndsystems.com/products/recombinant-mouse-dnam-1-cd226-fc-chimera-protein-cf_4436-dn#product-details
Biolegend	342606	APC anti-human HLA-E Antibody	1/100	20 min on ice	Each lot of this antibody is quality control tested by immunofluorescent staining with flow cytometric analysis by the manufacturer. See the manufacturer page for validation data and references:	https://www.biolegend.com/en-us/products/apc-anti-human-hla-e-antibody-10760
Biolegend	335917	Alexa Fluor® 488 anti-human HLA-G Antibody	1/100	20 min on ice	Each lot of this antibody is quality control tested by immunofluorescent staining with flow cytometric analysis by the manufacturer. See the manufacturer page for validation data and references:	https://www.biolegend.com/en-us/products/alexa-fluor-488-anti-human-hla-g-antibody-15424
Fisher	FAB5674P	Mouse L1CAM PE-conjugated Antibody	1/100	20 min on ice	Reactivity with mouse species was established.Detects mouse L1CAM in direct ELISAs. In direct ELISAs, no cross-reactivity with recombinant human (rh) ALCAM, rhBCAM, rhEPCAM, rhMCAM, rhNCAM, rhNCAM-L1, rhOBCAM, recombinant mouse (rm) MAdCAM-1, or rmOCAM is observed. For validation, Mouse splenocytes were stained with Rat Anti-Mouse L1CAM PE-conjugated Monoclonal Antibody (Catalog # FAB5674P, filled histogram) or isotype control antibody (IC006P, open histogram). See manufacturer's website for validation data:	https://www.fishersci.com/shop/products/anti-l1cam-pe-clone-555-r-d-systems/FAB5674P?searchHijack=true&searchTerm=FAB5674P&searchType=RAPID&matchedCatNo=FAB5674P
Biolegend	371604	PE anti-human CD171 (L1CAM) Antibody	1/100	20 min on ice	Each lot of this antibody is quality control tested by immunofluorescent staining with flow cytometric analysis by the manufacturer. See the manufacturer page for validation data and references:	https://www.biolegend.com/en-us/products/pe-anti-human-cd171-l1cam-antibody-13168
Biolegend	372806	APC anti-human CD133 Antibody	1/100	20 min on ice	Each lot of this antibody is quality control tested by immunofluorescent staining with flow cytometric analysis by the manufacturer. See the manufacturer page for validation data and references:	https://www.biolegend.com/en-us/products/apc-anti-human-cd133-antibody-13915
Biolegend	640906	FITC Annexin V	1/100	15 min at room temp	Each lot of this antibody is quality control tested by immunofluorescent staining with flow cytometric analysis by the manufacturer. See the manufacturer page for validation data and references:	https://www.biolegend.com/en-us/products/fitc-annexin-v-5161

BioLegend	125212	PerCP/Cy5.5 anti-mouse CD223 (LAG-3)	1/100	20 min on ice	Each lot of this antibody is quality control tested by immunofluorescent staining with flow cytometric analysis by the manufacturer. See the manufacturer page for validation data and references:	https://www.biolegend.com/en-us/products/percp-cyanine5-5-anti-mouse-cd223-lag-3-antibody-8141
BioLegend	119706	APC anti-mouse CD366 (Tim-3)	1/100	20 min on ice	Each lot of this antibody is quality control tested by immunofluorescent staining with flow cytometric analysis by the manufacturer. See the manufacturer page for validation data and references:	https://www.biolegend.com/en-us/products/apc-anti-mouse-cd366-tim-3-antibody-8238
BioLegend	101216	PE/Cyanine7 anti-mouse/human CD11b Antibody	1/100	20 min on ice	Each lot of this antibody is quality control tested by immunofluorescent staining with flow cytometric analysis by the manufacturer. See the manufacturer page for validation data and references:	https://www.biolegend.com/en-us/products/pe-cyanine7-anti-mouse-human-cd11b-antibody-1921
Biolegend	121419	FITC anti-mouse CD103 Antibody	1/100	20 min on ice	Each lot of this antibody is quality control tested by immunofluorescent staining with flow cytometric analysis by the manufacturer. See the manufacturer page for validation data and references:	https://www.biolegend.com/en-us/products/fitc-anti-mouse-cd103-antibody-7053
Biolegend	127618	PE/Cyanine7 anti-mouse Ly-6G Antibody	1/100	20 min on ice	Each lot of this antibody is quality control tested by immunofluorescent staining with flow cytometric analysis by the manufacturer. See the manufacturer page for validation data and references:	https://www.biolegend.com/en-us/products/pe-cyanine7-anti-mouse-ly-6g-antibody-6139
Biolegend	128008	PE anti-mouse Ly-6C Antibody	1/100	20 min on ice	Each lot of this antibody is quality control tested by immunofluorescent staining with flow cytometric analysis by the manufacturer. See the manufacturer page for validation data and references:	https://www.biolegend.com/en-us/products/pe-anti-mouse-ly-6c-antibody-4904
Biolegend	503808	PE anti-mouse IL-2 Antibody	1/100	20 min on ice	Each lot of this antibody is quality control tested by immunofluorescent staining with flow cytometric analysis by the manufacturer. See the manufacturer page for validation data and references:	https://www.biolegend.com/en-us/products/pe-anti-mouse-il-2-antibody-954
Biolegend	151208	Brilliant Violet 421™ anti-mouse/human Ki-67	1/100	20 min on ice	Each lot of this antibody is quality control tested by immunofluorescent staining with flow cytometric analysis by the manufacturer. See the manufacturer page for validation data and references:	https://www.biolegend.com/en-us/products/brilliant-violet-421-anti-mouse-human-ki-67-antibody-13639
R&D	139NK050	R&D Systems™ Mouse NKG2D/CD314 Fc Chimera Recombinant Protein	1/100	20 min on ice	Activity is measured by its binding ability in a functional ELISA. Immobilized rmNKG2D/Fc Chimera at 4 µg/mL (100 µL/well) can bind biotinylated rmRae-1 gamma /Fc Chimera with a linear range of 1.56-100 ng/mL. See manufacturer's website for validation data:	https://www.rndsystems.com/products/recombinant-mouse-nkg2d-fc-chimera-protein-cf_139-nk
Sinobiological	10575-H01S-50	Human NKG2D / CD314 / KLRK1 Protein (Fc Tag)	1/100	20 min on ice	HPLC-verified. See manufacturer's website for validation data:	https://www.sinobiological.com/recombinant-proteins/human-nkg2d-10575-h01s
Fisher	50-112-8857	Invitrogen™ eBioscience™ Foxp3 / Transcription Factor Staining Buffer Set	N/A	N/A	Buffer test is formulated and optimized for staining with antibodies against transcription factors and nuclear proteins (e.g. Foxp3 and Ki-67), cytokines and chemokines	https://www.fishersci.com/shop/products/foxp3-transcription-factor-staining-buffer-set/501128857?searchHijack=true&searchTerm=50-112-8857&searchType=RAPID&matchedCatNo=50-112-8857

In vivo Antibodies

BioXcell	BE0036	InVivoMAb anti-mouse NK1.1		Antibody has reported activity for in vivo NK depletion. NK cell depletion was confirmed in house with flow cytometry. See manufacturer's website for references:	https://bioxcell.com/invivomab-anti-mouse-nk1-1-be0036#tab_pdetails
BioXcell	BE0085	InVivoMAb mouse IgG2a isotype control, unknown specificity		See manufacturer's wesbite for more detailed information.	https://bioxcell.com/invivomab-mouse-igg2a-isotype-control-unknown-specificity-be0085#tab_specifications
BioXcell	BE0090	InVivoMAb rat IgG2b isotype control	Detailed in methods section.	See manufacturer's wesbite for more detailed information.	https://bioxcell.com/invivomab-rat-igg2b-isotype-control-anti-keyhole-limpet-hemocyanin-be0090
BioXcell	BE0003	In vivo MAb Anti-mouse CD4		Antibody has reported activity for in vivo CD4 depletion, flow cytometry and western blotting. CD4 depletion was confirmed in house by flow cytometry. See manufacturer's website for references:	https://bioxcell.com/invivomab-anti-mouse-cd4-be0003-1
BioXcell	BE0117	InVivoMAb anti-mouse CD8α		Antibody has reported activity for in vivo CD8 depletion and western blotting. CD8 depletion was confirmed in house by flow cytometry. See manufacturer's website for references:	https://bioxcell.com/invivomab-anti-mouse-cd8a-be0117

IHC and Western Blot

Abcam	ab211327	Recombinant Anti-MASH1/Achaete-scute homolog 1 antibody	1/100	4C Overnight	Antibody is tested for IHC and WB by the manufacturer. See manufacturer's website for testing data:	https://www.abcam.com/products/primary-antibodies/mash1achaete-scute-homolog-1-antibody-epr19840-ab211327.html
cell signaling technology	#9662	Caspase-3 Antibody	1/1000	4C Overnight	Antibody is tested for IHC and WB by the manufacturer. See manufacturer's website for testing data:	https://www.cellsignal.com/products/primary-antibodies/caspase-3-antibody/9662
cell signaling technology	2203T	TCF1/TCF7 (C63D9) Rabbit mAb	1/200	4C Overnight	Antibody is tested for IHC and WB by the manufacturer. See manufacturer's website for testing data:	https://www.cellsignal.com/products/primary-antibodies/tcf1-tcf7-c63d9-rabbit-mab/2203
Cell Signaling	9718S	Phospho-Histone H2A.X (Ser139) (20E3) Rabbit mAb	1/500	4C Overnight	Antibody is tested for IHC and WB by the manufacturer. See manufacturer's website for testing data:	https://www.cellsignal.com/products/primary-antibodies/phospho-histone-h2a-x-ser139-20e3-rabbit-mab/9718
Cell Signaling	5483S	Phospho-TBK1/NAK (Ser172) (D52C2) XP® Rabbit mAb	1/1000	4C Overnight	Antibody is tested for IHC and WB by the manufacturer. See manufacturer's website for testing data:	https://www.cellsignal.com/products/primary-antibodies/phospho-tbk1-nak-ser172-d52c2-xp-rabbit-mab/5483
Cell Signaling	3504S	TBK1/NAK (D1B4) Rabbit mAb	1/1000	4C Overnight	Antibody is tested for IHC and WB by the manufacturer. See manufacturer's website for testing data:	https://www.cellsignal.com/products/primary-antibodies/tbk1-nak-d1b4-rabbit-mab/3504
Cell Signaling Technology	4302	IRF-3 (D83B9) Rabbit mAb	1/1000	4C Overnight	Antibody is tested for IHC and WB by the manufacturer. See manufacturer's website for testing data:	https://www.cellsignal.com/products/primary-antibodies/irf-3-d83b9-rabbit-mab/4302
Cell Signaling Technology	4947	Phospho-IRF-3 (Ser396) (4D4G) Rabbit mAb	1/1000	4C Overnight	Antibody is tested for IHC and WB by the manufacturer. See manufacturer's website for testing data:	https://www.cellsignal.com/products/primary-antibodies/phospho-irf-3-ser396-4d4g-rabbit-mab/4947
Fisher	NC1651968	Cell Signaling Technology PHOSPHO-STING (SER365) (D8F4W)	1/1000	4C Overnight	Antibody is tested for IHC and WB by the manufacturer. See manufacturer's website for testing data:	https://www.cellsignal.com/products/primary-antibodies/tbk1-nak-d1b4-rabbit-mab/3504
Fisher	NC0665193	STING (D2P2F) Rabbit mAb	1/1000	4C Overnight	Antibody is tested for IHC and WB by the manufacturer. See manufacturer's website for testing data:	https://www.cellsignal.com/products/primary-antibodies/sting-d2p2f-rabbit-mab/13647
Cell Signaling	13901S	Vinculin (E1E9V) XP® Rabbit mAb #13901	1/1000	4C Overnight	Antibody is tested for IHC and WB by the manufacturer. See manufacturer's website for testing data:	https://www.cellsignal.com/products/primary-antibodies/vinculin-e1e9v-xp-rabbit-mab/13901
abcam	ab5690	Anti-CD3 antibody	1/100	4C Overnight	Antibody is tested for IHC and WB by the manufacturer. See manufacturer's website for testing data:	https://www.abcam.com/products/primary-antibodies/alpha-smooth-muscle-actin-antibody-ab5694.html
Fisher	MA5-14520	Ki-67 Recombinant Rabbit Monoclonal Antibody (SP6)	1/400	1hr room temperature	Antibody is tested for IHC and WB by the manufacturer. See manufacturer's website for testing data:	https://www.thermofisher.com/antibody/product/Ki-67-Antibody-clone-SP6-Recombinant-Monoclonal/MA5-14520

TIF Assay-Chrosome Spreads

PNA Bio	F3002	Cy3 conjugated CENPB (ATTCGTTGGAAACGGGA)	25ug/mL	4C Overnight	See the reference publications for the probe on manufacturer's website:	https://www.pnabio.com/products/PNA_FISH.htm
Panagene	F1002	Cy3-conjugated PNA Tel-C (CCCTAA)3 probe	25ug/mL	4C Overnight	Not provided for this product	http://www.panagene.com/_ENG/html/dh_product/prod_view/32/?cate_no=3
PNA Bio	F1008	Alexa 488-conjugated PNA Tel-G (TTAGGG)	25ug/mL	4C Overnight	See the reference publications for the probe on manufacturer's website:	https://www.pnabio.com/products/PNA_FISH.htm
Millipore	5636	Anti-phospho-Histone H2A.X (Ser139) Antibody, clone JBW301	1/200	4C Overnight	This Anti-phospho Histone H2A.X (Ser139) Antibody, clone JBW301, is validated for use in ICC for the detection of phospho Histone H2A.X (Ser139). See manufacturer's website for validation data.	https://www.emdmillipore.com/US/en/product/Anti-phospho-Histone-H2A.X-Ser139-Antibody-clone-JBW301-Alexa-Fluor-647,MM_NF-05-636-AF647
Santa Cruz	376248	Lamin A/C Antibody (E-1): sc-376248	1/200	4C Overnight	Lamin A/C Antibody (E-1) is a mouse monoclonal IgG1 κ Lamin A/C antibody is cited in 229 publications. See manufacturer's website for reference publications.	https://www.scbt.com/p/lamin-a-c-antibody-e-1?requestFrom=search

Table S1: Antibody information
