# Supplementary material for: A telomere-targeting drug depletes cancer initiating cells and promotes anti-tumor immunity in small cell lung cancer
Source: Nat Commun. 2024 Jan 22;15:672. doi: 10.1038/s41467-024-44861-8 (PMC10803750; doi:10.1038/s41467-024-44861-8)
Supplement: Supplementary file 3 — Reporting Summary [file 41467_2024_44861_MOESM3_ESM.pdf]

nature portfolio

Corresponding author(s): Esra Akbay, Ph.D.

Last updated by author(s): Dec 9, 2023

Reporting Summary

Nature Portfolio wishes to improve the reproducibility of the work that we publish. This form provides structure for consistency and transparency in reporting. For further information on Nature Portfolio policies, see our Editorial Policies and the Editorial Policy Checklist.

Statistics

For all statistical analyses, confirm that the following items are present in the figure legend, table legend, main text, or Methods section.

n/a	Confirmed	
☐	☒	The exact sample size (*n*) for each experimental group/condition, given as a discrete number and unit of measurement
☒	☐	A statement on whether measurements were taken from distinct samples or whether the same sample was measured repeatedly
☐	☒	The statistical test(s) used AND whether they are one- or two-sided<br>*Only common tests should be described solely by name; describe more complex techniques in the Methods section.*
☐	☒	A description of all covariates tested
☐	☒	A description of any assumptions or corrections, such as tests of normality and adjustment for multiple comparisons
☐	☒	A full description of the statistical parameters including central tendency (e.g. means) or other basic estimates (e.g. regression coefficient) AND variation (e.g. standard deviation) or associated estimates of uncertainty (e.g. confidence intervals)
☐	☒	For null hypothesis testing, the test statistic (e.g. *F*, *t*, *r*) with confidence intervals, effect sizes, degrees of freedom and *P* value noted<br>*Give P values as exact values whenever suitable.*
☐	☒	For Bayesian analysis, information on the choice of priors and Markov chain Monte Carlo settings
☐	☒	For hierarchical and complex designs, identification of the appropriate level for tests and full reporting of outcomes
☐	☒	Estimates of effect sizes (e.g. Cohen's *d*, Pearson's *r*), indicating how they were calculated

Our web collection on statistics for biologists contains articles on many of the points above.

Software and code

Policy information about availability of computer code

Data collection	BD FACSDiva v9.0 to collect flow cytometry data. NIS-ElementD 5.20.00 for all microscope images; QuantStudio Design&Analysis software for qPCR.
Data analysis	Graph pad Prism 10.1.1 for plots and statistical analysis. FlowJo (vl0.7) for analyzing flow cytometry data. Single cell sequencing was analyzed using Seurat package (v3). IHC staining was quantified using Image J or QuPath (v0.5.0)

For manuscripts utilizing custom algorithms or software that are central to the research but not yet described in published literature, software must be made available to editors and reviewers. We strongly encourage code deposition in a community repository (e.g. GitHub). See the Nature Portfolio guidelines for submitting code & software for further information.

Data

Policy information about availability of data

All manuscripts must include a data availability statement. This statement should provide the following information, where applicable:

- Accession codes, unique identifiers, or web links for publicly available datasets
- A description of any restrictions on data availability
- For clinical datasets or third party data, please ensure that the statement adheres to our policy

The scRNA-seq data was deposited as GEO accession GSE225018 and is publicly available.

Research involving human participants, their data, or biological material

Policy information about studies with human participants or human data. See also policy information about sex, gender (identity/presentation), and sexual orientation and race, ethnicity and racism.

Reporting on sex and gender	N/A because no human subjects in this article
Reporting on race, ethnicity, or other socially relevant groupings	N/A because no human subjects in this article
Population characteristics	N/A because no human subjects in this article
Recruitment	N/A because no human subjects in this article
Ethics oversight	N/A because no human subjects in this article

Note that full information on the approval of the study protocol must also be provided in the manuscript.

Field-specific reporting

Please select the one below that is the best fit for your research. If you are not sure, read the appropriate sections before making your selection.

[x] Life sciences [ ] Behavioural & social sciences [ ] Ecological, evolutionary & environmental sciences

For a reference copy of the document with all sections, see nature.com/documents/nr-reporting-summary-flat.pdf

Life sciences study design

All studies must disclose on these points even when the disclosure is negative.

Sample size	sample size was determined based on power analysis or availability of precious resources in the case of humanized mice.
Data exclusions	No data were excluded from analysis.
Replication	Experiments were repeated at least twice.
Randomization	Samples were all evenly distributed between experimental group based on biological variances such as age and sex.
Blinding	Blinding was not possible for group allocation because investigators allocate the subjects. Blinding was performed during data collection and data analysis.

Reporting for specific materials, systems and methods

We require information from authors about some types of materials, experimental systems and methods used in many studies. Here, indicate whether each material, system or method listed is relevant to your study. If you are not sure if a list item applies to your research, read the appropriate section before selecting a response.

Materials & experimental systems

n/a	Involved in the study	
[ ]	[x]	Antibodies
[ ]	[x]	Eukaryotic cell lines
[x]	[ ]	Palaeontology and archaeology
[ ]	[x]	Animals and other organisms
[x]	[ ]	Clinical data
[x]	[ ]	Dual use research of concern
[x]	[ ]	Plants

Methods

n/a	Involved in the study	
[x]	[ ]	ChIP-seq
[ ]	[x]	Flow cytometry
[x]	[ ]	MRI-based neuroimaging

Antibodies

Antibodies used

Antibody List
Flow Cytometry
Company Cat # Antibody
Biolegend 304006 Alexa Fluor® 488 anti-human CD45
Biolegend 320912 Alexa Fluor® 488 anti-human MICA/MICB

Biolegend 100423 Alexa Fluor® 488 anti-mouse CD4
Biolegend 103122 Alexa Fluor® 488 anti-mouse CD45
Biolegend 108913 Alexa Fluor® 488 anti-mouse CD49b (pan-NK cells)
Biolegend 100723 Alexa Fluor® 488 anti-mouse CD8a
Biolegend 100516 APC anti-mouse CD4
Biolegend 503810 APC anti-mouse IL-2
Biolegend 400612 APC Rat IgG2b, κ Isotype Ctrl
Biolegend 103116 APC/Cy7 anti-mouse CD45
Biolegend 100714 APC/Cy7 anti-mouse CD8a
Biolegend 115538 Brilliant Violet 421™ anti-mouse CD19
Biolegend 652411 Brilliant Violet 421™ anti-mouse Ki-67
Biolegend 118216 PE/Cy7 anti-mouse CD326 (Ep-CAM)
Biolegend 100320 PE/Cy7 anti-mouse CD3ε
Biolegend 324214 PerCP/Cy5.5 anti-human CD326 (Ep-CAM)
Biolegend 137610 PerCP/Cy5.5 anti-mouse CD335 (NKp46)
Biolegend 400233 Alexa Fluor® 488 Mouse IgG2a, κ Isotype Ctrl Antibody
Biolegend 400636 PE Rat IgG2b, κ Isotype Ctrl Antibody
Biolegend 400220 APC Mouse IgG2a, κ Isotype Ctrl Antibody
Biolegend 311410 APC anti-human HLA-A,B,C Antibody
Biolegend 137607 APC anti-mouse CD335 (NKp46) Antibody
Biolegend 114607 PE anti-mouse H-2K b/H-2D b Antibody
Biolegend 400212 PE Mouse IgG2a, κ Isotype Ctrl Antibody
Biolegend 400341 Brilliant Violet 421™ Mouse IgG2b, κ Isotype Ctrl Antibody
Biolegend 133203 PE anti-mouse RAE-1δ Antibody
Biolegend 400111 PE Mouse IgG1, κ Isotype Ctrl Antibody
Biolegend 400319 APC Mouse IgG2b, κ Isotype Ctrl Antibody
Biolegend 410711 APC anti-human IgG Fc Antibody
Biolegend 400511 APC Rat IgG2a, κ Isotype Ctrl Antibody
Biolegend 400119 APC Mouse IgG1, κ Isotype Ctrl Antibody
Biolegend 100733 PerCP/Cy5.5 anti-mouse CD8a Antibody
Biolegend 407110 APC anti-mouse IgG2a Antibody
Biolegend 400411 APC Rat IgG1, κ Isotype Ctrl Antibody
Biolegend 331913 Brilliant Violet 421™ anti-human CD335 (NKp46) Antibody
Biolegend 400157 Brilliant Violet 421™ Mouse IgG1, κ Isotype Ctrl Antibody
Biolegend 400125 PE/Cy7 Mouse IgG1, κ Isotype Ctrl Antibody
Biolegend 100328 PerCP/Cy5.5 anti-mouse CD3ε Antibody
Biolegend 100321 Alexa Fluor® 488 anti-mouse CD3ε Antibody
Biolegend 100512 PE anti-mouse CD4 Antibody
Biolegend 121614 APC anti-mouse CD107a (LAMP-1) Antibody
Biolegend 121626 PerCP/Cy5.5 anti-mouse CD107a (LAMP-1) Antibody
Biolegend 400549 Brilliant Violet 421™ Rat IgG2a, κ Isotype Ctrl Antibody
Biolegend 503808 PE anti-mouse IL-2 Antibody
Biolegend 101320 TruStain fcX™ (anti-mouse CD16/32) Antibody
Biolegend 100341 Brilliant Violet 421™ anti-mouse CD3ε Antibody
Biolegend 400408 PE Rat IgG1, κ Isotype Ctrl Antibody
Biolegend 400326 PE/Cy7 Mouse IgG2b, κ Isotype Ctrl Antibody
Biolegend 100320 PE/Cy7 anti-mouse CD3ε Antibody
Biolegend 400508 PE Rat IgG2a, κ Isotype Ctrl Antibody
Biolegend 311406 PE anti-human HLA-A,B,C Antibody
Biolegend 400214 PE Mouse IgG2a, κ Isotype Ctrl (FC) Antibody
Biolegend 114608 PE anti-mouse H-2Kb/H-2Db Antibody
Biolegend 503826 Brilliant Violet 421™ anti-mouse IL-2 Antibody
Biolegend 506510 APC anti-human IFN-γ Antibody
Biolegend 300316 PE/Cy7 anti-human CD3 Antibody
Biolegend 372206 FITC anti-human/mouse Granzyme B Recombinant Antibody
Biolegend 100734 PerCP/Cyanine5.5 anti-mouse CD8a Antibody
Biolegend 400138 FITC Mouse IgG1, κ Isotype Ctrl (ICFC) Antibody
Biolegend 108708 PE anti-mouse NK-1.1 Antibody
Biolegend 108913 Alexa Fluor® 488 anti-mouse CD49b (pan-NK cells) Antibody
Biolegend 108910 APC anti-mouse CD49b (pan-NK cells) Antibody
Biolegend 400522 PE/Cy7 Rat IgG2a, κ Isotype Ctrl Antibody
Biolegend 400617 PE/Cy7 Rat IgG2b, κ Isotype Ctrl Antibody
Biolegend 324221 PE/Cy7 anti-human CD326 (EpCAM) Antibody
Biolegend 304014 APC/Cyanine7 anti-human CD45 Antibody
Biolegend 320906 PE anti-human MICA/MICB Antibody
Biolegend 505808 PE anti-mouse IFN-γ Antibody
Biolegend 103116 APC/Cyanine7 anti-mouse CD45 Antibody
R&D 666-DN Human DNAM-1/CD226 Fc Chimera Recombinant Protein

R&D 4436-DN Mouse DNAM-1/CD226 Fc Chimera Recombinant Protein
Biolegend 342606 APC anti-human HLA-E Antibody
Biolegend 335917 Alexa Fluor® 488 anti-human HLA-G Antibody
Fisher FAB5674P Mouse L1CAM PE-conjugated Antibody
Biolegend 371604 PE anti-human CD171 (L1CAM) Antibody
Biolegend 372806 APC anti-human CD133 Antibody
Biolegend 640906 FITC Annexin V
BioLegend 125212 PerCP/Cy5.5 anti-mouse CD223 (LAG-3)
BioLegend 119706 APC anti-mouse CD366 (Tim-3)
BioLegend 101216 PE/Cyanine7 anti-mouse/human CD11b Antibody
Biolegend 121419 FITC anti-mouse CD103 Antibody
Biolegend 127618 PE/Cyanine7 anti-mouse Ly-6G Antibody
Biolegend 128008 PE anti-mouse Ly-6C Antibody
Biolegend 503808 PE anti-mouse IL-2 Antibody
Biolegend 151208 Brilliant Violet 421™ anti-mouse/human Ki-67 Antibody
R&D 139NK050 R&D Systems™ Mouse NKG2D/CD314 Fc Chimera Recombinant Protein
Sinobiological 10575-H01S-50 Human NKG2D / CD314 / KLRK1 Protein (Fc Tag)
Fisher 50-112-8857 Invitrogen™ eBioscience™ Foxp3 / Transcription Factor Staining Buffer Set
In vivo Antibodies
BioXcell BE0036 InVivoMAb anti-mouse NK1.1
BioXcell BE0085 InVivoMAb mouse IgG2a isotype control, unknown specificity
BioXcell BE0090 InVivoMAb rat IgG2b isotype control
BioXcell BE0003 In vivo MAb Anti-mouse CD4
BioXcell BE0117 InVivoMAb anti-mouse CD8α
IHC and Western Blot
Abcam ab211327 Recombinant Anti-MASH1/Achaete-scute homolog 1 antibody
cell signaling technology #9662 Caspase-3 Antibody
cell signaling technology 2203T TCF1/TCF7 (C63D9) Rabbit mAb
Cell Signaling 9718S Phospho-Histone H2A.X (Ser139) (20E3) Rabbit mAb
Cell Signaling 5483S Phospho-TBK1/NAK (Ser172) (D52C2) XP® Rabbit mAb
Cell Signaling 3504S TBK1/NAK (D1B4) Rabbit mAb
Cell Signaling Technology 4302 IRF-3 (D83B9) Rabbit mAb
Cell Signaling Technology 4947 Phospho-IRF-3 (Ser396) (4D4G) Rabbit mAb
Fisher NC1651968 Cell Signaling Technology PHOSPHO-STING (SER365) (D8F4W)
Fisher NC0665193 STING (D2P2F) Rabbit mAb
Cell Signaling 13901S Vinculin (E1E9V) XP® Rabbit mAb #13901
abcam ab5690 Anti-CD3 antibody
Fisher MA5-14520 Ki-67 Recombinant Rabbit Monoclonal Antibody (SP6)
TIF Assay-Chrosome Spreads
PNA Bio F3002 Cy3 conjugated CENPB (ATTCGTTGGAAACGGGA)
Panagene F1002 Cy3-conjugated PNA Tel-C (CCCTAA)3 probe
PNA Bio F1008 Alexa 488-conjugated PNA Tel-G (TTAGGG)
Millipore 5636 Anti-phospho-Histone H2A.X (Ser139) Antibody, clone JBW301
Santa Cruz 376248 Lamin A/C Antibody (E-1): sc-376248

Validation

Antibody List
Flow Cytometry
Company Cat # Antibody Validation
Biolegend 304006 Alexa Fluor® 488 anti-human CD45 Each lot of this antibody is quality control tested by immunofluorescent staining with flow cytometric analysis by the manufacturer. See the manufacturer page for validation data and references: https://www.biolegend.com/en-us/products/fitc-anti-human-cd45-antibody-707
Biolegend 320912 Alexa Fluor® 488 anti-human MICA/MICB Each lot of this antibody is quality control tested by immunofluorescent staining with flow cytometric analysis by the manufacturer. See the manufacturer page for validation data and references: https://www.biolegend.com/en-us/products/alexa-fluor-488-anti-human-mica-micb-antibody-3067
Biolegend 100423 Alexa Fluor® 488 anti-mouse CD4 Each lot of this antibody is quality control tested by immunofluorescent staining with flow cytometric analysis by the manufacturer. See the manufacturer page for validation data and references: https://www.biolegend.com/en-us/products/alexa-fluor-488-anti-mouse-cd4-antibody-2695
Biolegend 103122 Alexa Fluor® 488 anti-mouse CD45 Each lot of this antibody is quality control tested by immunofluorescent staining with flow cytometric analysis by the manufacturer. See the manufacturer page for validation data and references: https://www.biolegend.com/en-us/products/alexa-fluor-488-anti-mouse-cd45-antibody-3100
Biolegend 108913 Alexa Fluor® 488 anti-mouse CD49b (pan-NK cells) Each lot of this antibody is quality control tested by immunofluorescent staining with flow cytometric analysis by the manufacturer. See the manufacturer page for validation data and references: https://www.biolegend.com/en-us/products/alexa-fluor-488-anti-mouse-cd49b-pan-nk-cells-antibody-2709
Biolegend 100723 Alexa Fluor® 488 anti-mouse CD8a Each lot of this antibody is quality control tested by immunofluorescent staining with flow cytometric analysis by the manufacturer. See the manufacturer page for validation data and references: https://www.biolegend.com/en-us/products/alexa-fluor-488-anti-mouse-cd8a-antibody-2698
Biolegend 100516 APC anti-mouse CD4 Each lot of this antibody is quality control tested by immunofluorescent staining with flow cytometric analysis by the manufacturer. See the manufacturer page for validation data and references: https://www.biolegend.com/en-us/products/apc-anti-mouse-cd4-antibody-477
Biolegend 503810 APC anti-mouse IL-2 Each lot of this antibody is quality control tested by immunofluorescent staining with flow

cytometric analysis by the manufacturer. See the manufacturer page for validation data and references: https://www.biolegend.com/en-us/products/apc-anti-mouse-il-2-antibody-950
Biolegend 400612 APC Rat IgG2b, κ Isotype Ctrl Each lot of this antibody is quality control tested by immunofluorescent staining with flow cytometric analysis as negative control by the manufacturer. See the manufacturer page for validation data and references: https://www.biolegend.com/en-us/products/apc-rat-igg2b-kappa-isotype-ctrl-1851
Biolegend 103116 APC/Cy7 anti-mouse CD45 Each lot of this antibody is quality control tested by immunofluorescent staining with flow cytometric analysis by the manufacturer. See the manufacturer page for validation data and references: https://www.biolegend.com/en-us/products/apc-cyanine7-anti-mouse-cd45-antibody-2530
Biolegend 100714 APC/Cy7 anti-mouse CD8a Each lot of this antibody is quality control tested by immunofluorescent staining with flow cytometric analysis by the manufacturer. See the manufacturer page for validation data and references: https://www.biolegend.com/en-us/products/apc-cyanine7-anti-mouse-cd8a-antibody-2269
Biolegend 115538 Brilliant Violet 421™ anti-mouse CD19 Each lot of this antibody is quality control tested by immunofluorescent staining with flow cytometric analysis by the manufacturer. See the manufacturer page for validation data and references: https://www.biolegend.com/en-us/products/brilliant-violet-421-anti-mouse-cd19-antibody-7160
Biolegend 652411 Brilliant Violet 421™ anti-mouse Ki-67 Each lot of this antibody is quality control tested by immunofluorescent staining with flow cytometric analysis by the manufacturer. See the manufacturer page for validation data and references: https://www.biolegend.com/en-us/products/brilliant-violet-421-anti-mouse-ki-67-antibody-8982
Biolegend 118216 PE/Cy7 anti-mouse CD326 (Ep-CAM) Each lot of this antibody is quality control tested by immunofluorescent staining with flow cytometric analysis by the manufacturer. See the manufacturer page for validation data and references: https://www.biolegend.com/en-us/products/pe-cyanine7-anti-mouse-cd326-ep-cam-antibody-5303
Biolegend 100320 PE/Cy7 anti-mouse CD3ε Each lot of this antibody is quality control tested by immunofluorescent staining with flow cytometric analysis by the manufacturer. See the manufacturer page for validation data and references: https://www.biolegend.com/en-us/products/pe-cyanine7-anti-mouse-cd3epsilon-antibody-1899
Biolegend 324214 PerCP/Cy5.5 anti-human CD326 (Ep-CAM) Each lot of this antibody is quality control tested by immunofluorescent staining with flow cytometric analysis by the manufacturer. See the manufacturer page for validation data and references: https://www.biolegend.com/en-us/products/percp-cyanine5-5-anti-human-cd326-epcam-antibody-4252
Biolegend 137610 PerCP/Cy5.5 anti-mouse CD335 (NKp46) Each lot of this antibody is quality control tested by immunofluorescent staining with flow cytometric analysis by the manufacturer. See the manufacturer page for validation data and references: https://www.biolegend.com/en-us/products/percp-cyanine5-5-anti-mouse-cd335-nkp46-antibody-6724
Biolegend 400233 Alexa Fluor® 488 Mouse IgG2a, κ Isotype Ctrl Antibody Each lot of this antibody is quality control tested by immunofluorescent staining with flow cytometric analysis by the manufacturer. See the manufacturer page for validation data and references: https://www.biolegend.com/en-us/products/alexa-fluor-488-mouse-igg2a-kappa-isotype-ctrl-2690
Biolegend 400636 PE Rat IgG2b, κ Isotype Ctrl Antibody Each lot of this antibody is quality control tested by immunofluorescent staining with flow cytometric analysis by the manufacturer. See the manufacturer page for validation data and references: https://www.biolegend.com/en-us/products/pe-rat-igg2b-kappa-isotype-ctrl-1856
Biolegend 400220 APC Mouse IgG2a, κ Isotype Ctrl Antibody Each lot of this antibody is quality control tested by immunofluorescent staining with flow cytometric analysis by the manufacturer. See the manufacturer page for validation data and references: https://www.biolegend.com/en-us/products/apc-mouse-igg2a-kappa-isotype-ctrl-1397
Biolegend 311410 APC anti-human HLA-A,B,C Antibody Each lot of this antibody is quality control tested by immunofluorescent staining with flow cytometric analysis by the manufacturer. See the manufacturer page for validation data and references: https://www.biolegend.com/en-us/products/apc-anti-human-hla-a-b-c-antibody-1870
Biolegend 137607 APC anti-mouse CD335 (NKp46) Antibody Each lot of this antibody is quality control tested by immunofluorescent staining with flow cytometric analysis by the manufacturer. See the manufacturer page for validation data and references: https://www.biolegend.com/en-us/products/apc-anti-mouse-cd335-nkp46-antibody-6676
Biolegend 114607 PE anti-mouse H-2K b/H-2D b Antibody Each lot of this antibody is quality control tested by immunofluorescent staining with flow cytometric analysis by the manufacturer. See the manufacturer page for validation data and references: https://www.biolegend.com/en-us/products/pe-anti-mouse-h-2k-b-h-2d-b-antibody-1686
Biolegend 400212 PE Mouse IgG2a, κ Isotype Ctrl Antibody Each lot of this antibody is quality control tested by immunofluorescent staining with flow cytometric analysis as negative control by the manufacturer. See the manufacturer page for validation data and references: https://www.biolegend.com/en-us/products/pe-mouse-igg2a-kappa-isotype-ctrl-1401
Biolegend 400341 Brilliant Violet 421™ Mouse IgG2b, κ Isotype Ctrl Antibody Each lot of this antibody is quality control tested by immunofluorescent staining with flow cytometric analysis as negative control by the manufacturer. See the manufacturer page for validation data and references: https://www.biolegend.com/en-us/products/brilliant-violet-421-mouse-igg2b-kappa-isotype-ctrl-7195
Biolegend 133203 PE anti-mouse RAE-1δ Antibody Each lot of this antibody is quality control tested by immunofluorescent staining with flow cytometric analysis by the manufacturer. See the manufacturer page for validation data and references: https://www.biolegend.com/en-us/products/pe-anti-mouse-rae-1delta-antibody-5768
Biolegend 400111 PE Mouse IgG1, κ Isotype Ctrl Antibody Each lot of this antibody is quality control tested by immunofluorescent staining with flow cytometric analysis as negative control by the manufacturer. See the manufacturer page for validation data and references: https://www.biolegend.com/en-us/products/pe-mouse-igg1-kappa-isotype-ctrl-1408
Biolegend 400319 APC Mouse IgG2b, κ Isotype Ctrl Antibody Each lot of this antibody is quality control tested by immunofluorescent staining with flow cytometric analysis as negative control by the manufacturer. See the manufacturer page for validation data and references: https://www.biolegend.com/en-us/products/apc-mouse-igg2b-kappa-isotype-ctrl-1410
Biolegend 410711 APC anti-human IgG Fc Antibody Each lot of this antibody is quality control tested by immunofluorescent staining with flow cytometric analysis by the manufacturer. See the manufacturer page for validation data and references: https://www.biolegend.com/en-us/products/apc-anti-human-igg-fc-11935
Biolegend 400511 APC Rat IgG2a, κ Isotype Ctrl Antibody Each lot of this antibody is quality control tested by immunofluorescent staining with flow cytometric analysis as negative control by the manufacturer. See the manufacturer page for validation data and references: https://www.biolegend.com/en-us/products/apc-rat-igg2a-kappa-isotype-ctrl-1838
Biolegend 400119 APC Mouse IgG1, κ Isotype Ctrl Antibody Each lot of this antibody is quality control tested by immunofluorescent staining with flow cytometric analysis as negative control by the manufacturer. See the manufacturer page for validation data and references: https://www.biolegend.com/en-us/products/apc-mouse-igg1-kappa-isotype-ctrl-1404
Biolegend 100733 PerCP/Cy5.5 anti-mouse CD8a Antibody Each lot of this antibody is quality control tested by immunofluorescent

staining with flow cytometric analysis by the manufacturer. See the manufacturer page for validation data and references: https://www.biolegend.com/en-us/products/percp-cyanine5-5-anti-mouse-cd8a-antibody-4255
Biolegend 407110 APC anti-mouse IgG2a Antibody Each lot of this antibody is quality control tested by immunofluorescent staining with flow cytometric analysis by the manufacturer. See the manufacturer page for validation data and references: https://www.biolegend.com/en-us/products/apc-anti-mouse-igg2a-8327
Biolegend 400411 APC Rat IgG1, κ Isotype Ctrl Antibody Each lot of this antibody is quality control tested by immunofluorescent staining with flow cytometric analysis as negative control by the manufacturer. See the manufacturer page for validation data and references: https://www.biolegend.com/en-us/products/apc-rat-igg1-kappa-isotype-ctrl-1826
Biolegend 331913 Brilliant Violet 421™ anti-human CD335 (NKp46) Antibody Each lot of this antibody is quality control tested by immunofluorescent staining with flow cytometric analysis by the manufacturer. See the manufacturer page for validation data and references: https://www.biolegend.com/en-us/products/brilliant-violet-421-anti-human-cd335-nkp46-antibody-7529
Biolegend 400157 Brilliant Violet 421™ Mouse IgG1, κ Isotype Ctrl Antibody Each lot of this antibody is quality control tested by immunofluorescent staining with flow cytometric analysis as negative control by the manufacturer. See the manufacturer page for validation data and references: https://www.biolegend.com/en-us/products/brilliant-violet-421-mouse-igg1-kappa-isotype-ctrl-7194
Biolegend 400125 PE/Cy7 Mouse IgG1, κ Isotype Ctrl Antibody Each lot of this antibody is quality control tested by immunofluorescent staining with flow cytometric analysis as negative control by the manufacturer. See the manufacturer page for validation data and references: https://www.biolegend.com/en-us/products/pe-cyanine7-mouse-igg1-kappa-isotype-ctrl-1926
Biolegend 100328 PerCP/Cy5.5 anti-mouse CD3ε Antibody Each lot of this antibody is quality control tested by immunofluorescent staining with flow cytometric analysis by the manufacturer. See the manufacturer page for validation data and references: https://www.biolegend.com/en-us/products/percp-cyanine5-5-anti-mouse-cd3epsilon-antibody-4191
Biolegend 100321 Alexa Fluor® 488 anti-mouse CD3ε Antibody Each lot of this antibody is quality control tested by immunofluorescent staining with flow cytometric analysis by the manufacturer. See the manufacturer page for validation data and references: https://www.biolegend.com/en-us/products/alexa-fluor-488-anti-mouse-cd3epsilon-antibody-2676
Biolegend 100512 PE anti-mouse CD4 Antibody Each lot of this antibody is quality control tested by immunofluorescent staining with flow cytometric analysis by the manufacturer. See the manufacturer page for validation data and references: https://www.biolegend.com/en-us/products/pe-anti-mouse-cd4-antibody-482
Biolegend 121614 APC anti-mouse CD107a (LAMP-1) Antibody Each lot of this antibody is quality control tested by immunofluorescent staining with flow cytometric analysis by the manufacturer. See the manufacturer page for validation data and references: https://www.biolegend.com/en-us/products/apc-anti-mouse-cd107a-lamp-1-antibody-6081
Biolegend 121626 PerCP/Cy5.5 anti-mouse CD107a (LAMP-1) Antibody Each lot of this antibody is quality control tested by immunofluorescent staining with flow cytometric analysis by the manufacturer. See the manufacturer page for validation data and references: https://www.biolegend.com/en-us/products/percp-cyanine5-5-anti-mouse-cd107a-lamp-1-antibody-13079
Biolegend 400549 Brilliant Violet 421™ Rat IgG2a, κ Isotype Ctrl Antibody Each lot of this antibody is quality control tested by immunofluorescent staining with flow cytometric analysis as negative control by the manufacturer. See the manufacturer page for validation data and references: https://www.biolegend.com/en-us/products/brilliant-violet-421-rat-igg2a-kappa-isotype-ctrl-7135
Biolegend 503808 PE anti-mouse IL-2 Antibody Each lot of this antibody is quality control tested by immunofluorescent staining with flow cytometric analysis by the manufacturer. See the manufacturer page for validation data and references: https://www.biolegend.com/en-us/products/pe-anti-mouse-il-2-antibody-954
Biolegend 101320 TruStain fcX™ (anti-mouse CD16/32) Antibody Each lot of this antibody is quality control tested by immunofluorescent staining with flow cytometric analysis by the manufacturer. See the manufacturer page for validation data and references: https://www.biolegend.com/en-us/products/trustain-fcx-anti-mouse-cd16-32-antibody-5683
Biolegend 100341 Brilliant Violet 421™ anti-mouse CD3ε Antibody Each lot of this antibody is quality control tested by immunofluorescent staining with flow cytometric analysis by the manufacturer. See the manufacturer page for validation data and references: https://www.biolegend.com/en-us/products/brilliant-violet-421-anti-mouse-cd3epsilon-antibody-7132
Biolegend 400408 PE Rat IgG1, κ Isotype Ctrl Antibody Each lot of this antibody is quality control tested by immunofluorescent staining with flow cytometric analysis as negative control by the manufacturer. See the manufacturer page for validation data and references: https://www.biolegend.com/en-us/products/pe-rat-igg1-kappa-isotype-ctrl-1830
Biolegend 400326 PE/Cy7 Mouse IgG2b, κ Isotype Ctrl Antibody Each lot of this antibody is quality control tested by immunofluorescent staining with flow cytometric analysis as negative control by the manufacturer. See the manufacturer page for validation data and references: https://www.biolegend.com/en-us/products/pe-cyanine7-mouse-igg2b-kappa-isotype-ctrl-1928
Biolegend 100320 PE/Cy7 anti-mouse CD3ε Antibody Each lot of this antibody is quality control tested by immunofluorescent staining with flow cytometric analysis by the manufacturer. See the manufacturer page for validation data and references: https://www.biolegend.com/en-us/products/pe-cyanine7-anti-mouse-cd3epsilon-antibody-1899
Biolegend 400508 PE Rat IgG2a, κ Isotype Ctrl Antibody Each lot of this antibody is quality control tested by immunofluorescent staining with flow cytometric analysis as negative control by the manufacturer. See the manufacturer page for validation data and references: https://www.biolegend.com/en-us/products/pe-rat-igg2a-kappa-isotype-ctrl-1843
Biolegend 311406 PE anti-human HLA-A,B,C Antibody Each lot of this antibody is quality control tested by immunofluorescent staining with flow cytometric analysis by the manufacturer. See the manufacturer page for validation data and references: https://www.biolegend.com/en-us/products/pe-anti-human-hla-a-b-c-antibody-1872
Biolegend 400214 PE Mouse IgG2a, κ Isotype Ctrl (FC) Antibody Each lot of this antibody is quality control tested by immunofluorescent staining with flow cytometric analysis as negative control by the manufacturer. See the manufacturer page for validation data and references: https://www.biolegend.com/en-us/products/pe-mouse-igg2a-kappa-isotype-ctrl-fc-3043
Biolegend 114608 PE anti-mouse H-2Kb/H-2Db Antibody Each lot of this antibody is quality control tested by immunofluorescent staining with flow cytometric analysis by the manufacturer. See the manufacturer page for validation data and references: https://www.biolegend.com/en-us/products/pe-anti-mouse-h-2k-b-h-2d-b-antibody-1686
Biolegend 503826 Brilliant Violet 421™ anti-mouse IL-2 Antibody Each lot of this antibody is quality control tested by immunofluorescent staining with flow cytometric analysis by the manufacturer. See the manufacturer page for validation data and references: https://www.biolegend.com/en-us/products/brilliant-violet-421-anti-mouse-il-2-antibody-7202
Biolegend 506510 APC anti-human IFN-γ Antibody Each lot of this antibody is quality control tested by immunofluorescent staining with flow cytometric analysis by the manufacturer. See the manufacturer page for validation data and references: https://www.biolegend.com/en-us/products/apc-anti-human-ifn-gamma-antibody-1533
Biolegend 300316 PE/Cy7 anti-human CD3 Antibody Each lot of this antibody is quality control tested by immunofluorescent staining with flow cytometric analysis by the manufacturer. See the manufacturer page for validation data and references: https://

www.biolegend.com/en-us/products/pe-cyanine7-anti-human-cd3-antibody-1913
Biolegend 372206 FITC anti-human/mouse Granzyme B Recombinant Antibody Each lot of this antibody is quality control tested by immunofluorescent staining with flow cytometric analysis by the manufacturer. See the manufacturer page for validation data and references: https://www.biolegend.com/en-us/products/fitc-anti-human-mouse-granzyme-b-recombinant-antibody-14430
Biolegend 100734 PerCP/Cyanine5.5 anti-mouse CD8a Antibody Each lot of this antibody is quality control tested by immunofluorescent staining with flow cytometric analysis by the manufacturer. See the manufacturer page for validation data and references: https://www.biolegend.com/en-us/products/percp-cyanine5-5-anti-mouse-cd8a-antibody-4255
Biolegend 400138 FITC Mouse IgG1, κ Isotype Ctrl (ICFC) Antibody Each lot of this antibody is quality control tested by immunofluorescent staining with flow cytometric analysis as negative control by the manufacturer. See the manufacturer page for validation data and references: https://www.biolegend.com/en-us/products/fitc-mouse-igg1-kappa-isotype-ctrl-icfc-3031
Biolegend 108708 PE anti-mouse NK-1.1 Antibody Each lot of this antibody is quality control tested by immunofluorescent staining with flow cytometric analysis by the manufacturer. See the manufacturer page for validation data and references: https://www.biolegend.com/en-us/products/pe-anti-mouse-nk-1-1-antibody-431
Biolegend 108913 Alexa Fluor® 488 anti-mouse CD49b (pan-NK cells) Antibody Each lot of this antibody is quality control tested by immunofluorescent staining with flow cytometric analysis by the manufacturer. See the manufacturer page for validation data and references: https://www.biolegend.com/en-us/products/alexa-fluor-488-anti-mouse-cd49b-pan-nk-cells-antibody-2709
Biolegend 108910 APC anti-mouse CD49b (pan-NK cells) Antibody Each lot of this antibody is quality control tested by immunofluorescent staining with flow cytometric analysis by the manufacturer. See the manufacturer page for validation data and references: https://www.biolegend.com/en-us/products/apc-anti-mouse-cd49b-pan-nk-cells-antibody-231
Biolegend 400522 PE/Cy7 Rat IgG2a, κ Isotype Ctrl Antibody Each lot of this antibody is quality control tested by immunofluorescent staining with flow cytometric analysis as negative control by the manufacturer. See the manufacturer page for validation data and references: https://www.biolegend.com/en-us/products/pe-cyanine7-rat-igg2a-kappa-isotype-ctrl-1935
Biolegend 400617 PE/Cy7 Rat IgG2b, κ Isotype Ctrl Antibody Each lot of this antibody is quality control tested by immunofluorescent staining with flow cytometric analysis as negative control by the manufacturer. See the manufacturer page for validation data and references: https://www.biolegend.com/en-us/products/pe-cyanine7-rat-igg2b-kappa-isotype-ctrl-1936
Biolegend 324221 PE/Cy7 anti-human CD326 (EpCAM) Antibody Each lot of this antibody is quality control tested by immunofluorescent staining with flow cytometric analysis by the manufacturer. See the manufacturer page for validation data and references: https://www.biolegend.com/en-us/products/pe-cyanine7-anti-human-cd326-epcam-antibody-8107
Biolegend 304014 APC/Cyanine7 anti-human CD45 Antibody Each lot of this antibody is quality control tested by immunofluorescent staining with flow cytometric analysis by the manufacturer. See the manufacturer page for validation data and references: https://www.biolegend.com/en-us/products/apc-cyanine7-anti-human-cd45-antibody-1914
Biolegend 320906 PE anti-human MICA/MICB Antibody Each lot of this antibody is quality control tested by immunofluorescent staining with flow cytometric analysis by the manufacturer. See the manufacturer page for validation data and references: https://www.biolegend.com/en-us/products/pe-anti-human-mica-micb-antibody-3064
Biolegend 505808 PE anti-mouse IFN-γ Antibody Each lot of this antibody is quality control tested by immunofluorescent staining with flow cytometric analysis by the manufacturer. See the manufacturer page for validation data and references: https://www.biolegend.com/en-us/products/pe-anti-mouse-ifn-gamma-antibody-997
Biolegend 103116 APC/Cyanine7 anti-mouse CD45 Antibody Each lot of this antibody is quality control tested by immunofluorescent staining with flow cytometric analysis by the manufacturer. See the manufacturer page for validation data and references: https://www.biolegend.com/en-us/products/apc-cyanine7-anti-mouse-cd45-antibody-2530
R&D 666-DN Human DNAM-1/CD226 Fc Chimera Recombinant Protein Activity is measured by its binding ability in a functional ELISA.When Recombinant Human DNAM-1 Fc Chimera (Catalog # 666-DN) is immobilized at 1 µg/mL, 100 µL/well, Biotinylated Recombinant Human CD155/PVR (Catalog # BT9174) binds with an ED50 of 3-18 ng/mL. See manufacturer's website for validation information: https://www.rndsystems.com/products/recombinant-human-dnam-1-cd226-fc-chimera-protein-cf_666-dn#product-datasheets
R&D 4436-DN Mouse DNAM-1/CD226 Fc Chimera Recombinant Protein Activity is measured by its binding ability in a functional ELISA.When Recombinant Mouse Nectin-2/ CD112 (Catalog # 3869-N2) is coated at 1 µg/mL, Recombinant Mouse DNAM-1/CD226 Fc Chimera (Catalog # 4436-DN) binds with a typical ED50 of 0.3-1.8 µg/mL.See manufacturer's website for validation information: https://www.rndsystems.com/products/recombinant-mouse-dnam-1-cd226-fc-chimera-protein-cf_4436-dn#product-details
Biolegend 342606 APC anti-human HLA-E Antibody Each lot of this antibody is quality control tested by immunofluorescent staining with flow cytometric analysis by the manufacturer. See the manufacturer page for validation data and references: https://www.biolegend.com/en-us/products/apc-anti-human-hla-e-antibody-10760
Biolegend 335917 Alexa Fluor® 488 anti-human HLA-G Antibody Each lot of this antibody is quality control tested by immunofluorescent staining with flow cytometric analysis by the manufacturer. See the manufacturer page for validation data and references: https://www.biolegend.com/en-us/products/alexa-fluor-488-anti-human-hla-g-antibody-15424
Fisher FAB5674P Mouse L1CAM PE-conjugated Antibody Reactivity with mouse species was established.Detects mouse L1CAM in direct ELISAs. In direct ELISAs, no cross-reactivity with recombinant human (rh) ALCAM, rhBCAM, rhEPCAM, rhMCAM, rhNCAM, rhNCAM-L1, rhOBCAM, recombinant mouse (rm) MAdCAM-1, or rmOCAM is observed. For validation, Mouse splenocytes were stained with Rat Anti-Mouse L1CAM PE-conjugated Monoclonal Antibody (Catalog # FAB5674P, filled histogram) or isotype control antibody (IC006P, open histogram). See manufacturer's website for validation data: https://www.fishersci.com/shop/products/anti-l1cam-pe-clone-555-r-d-systems/FAB5674P?searchHijack=true&searchTerm=FAB5674P&searchType=RAPID&matchedCatNo=FAB5674P
Biolegend 371604 PE anti-human CD171 (L1CAM) Antibody Each lot of this antibody is quality control tested by immunofluorescent staining with flow cytometric analysis by the manufacturer. See the manufacturer page for validation data and references: https://www.biolegend.com/en-us/products/pe-anti-human-cd171-l1cam-antibody-13168
Biolegend 372806 APC anti-human CD133 Antibody Each lot of this antibody is quality control tested by immunofluorescent staining with flow cytometric analysis by the manufacturer. See the manufacturer page for validation data and references: https://www.biolegend.com/en-us/products/apc-anti-human-cd133-antibody-13915
Biolegend 640906 FITC Annexin V Each lot of this antibody is quality control tested by immunofluorescent staining with flow cytometric analysis by the manufacturer. See the manufacturer page for validation data and references: https://www.biolegend.com/en-us/products/fitc-annexin-v-5161
BioLegend 125212 PerCP/Cy5.5 anti-mouse CD223 (LAG-3) Each lot of this antibody is quality control tested by immunofluorescent staining with flow cytometric analysis by the manufacturer. See the manufacturer page for validation data and references: https://

www.biolegend.com/en-us/products/percp-cyanine5-5-anti-mouse-cd223-lag-3-antibody-8141
BioLegend 119706 APC anti-mouse CD366 (Tim-3) Each lot of this antibody is quality control tested by immunofluorescent staining with flow cytometric analysis by the manufacturer. See the manufacturer page for validation data and references: https://www.biolegend.com/en-us/products/apc-anti-mouse-cd366-tim-3-antibody-8238
BioLegend 101216 PE/Cyanine7 anti-mouse/human CD11b Antibody Each lot of this antibody is quality control tested by immunofluorescent staining with flow cytometric analysis by the manufacturer. See the manufacturer page for validation data and references: https://www.biolegend.com/en-us/products/pe-cyanine7-anti-mouse-human-cd11b-antibody-1921
Biolegend 121419 FITC anti-mouse CD103 Antibody Each lot of this antibody is quality control tested by immunofluorescent staining with flow cytometric analysis by the manufacturer. See the manufacturer page for validation data and references: https://www.biolegend.com/en-us/products/fitc-anti-mouse-cd103-antibody-7053
Biolegend 127618 PE/Cyanine7 anti-mouse Ly-6G Antibody Each lot of this antibody is quality control tested by immunofluorescent staining with flow cytometric analysis by the manufacturer. See the manufacturer page for validation data and references: https://www.biolegend.com/en-us/products/pe-cyanine7-anti-mouse-ly-6g-antibody-6139
Biolegend 128008 PE anti-mouse Ly-6C Antibody Each lot of this antibody is quality control tested by immunofluorescent staining with flow cytometric analysis by the manufacturer. See the manufacturer page for validation data and references: https://www.biolegend.com/en-us/products/pe-anti-mouse-ly-6c-antibody-4904
Biolegend 503808 PE anti-mouse IL-2 Antibody Each lot of this antibody is quality control tested by immunofluorescent staining with flow cytometric analysis by the manufacturer. See the manufacturer page for validation data and references: https://www.biolegend.com/en-us/products/pe-anti-mouse-il-2-antibody-954
Biolegend 151208 Brilliant Violet 421™ anti-mouse/human Ki-67 Antibody Each lot of this antibody is quality control tested by immunofluorescent staining with flow cytometric analysis by the manufacturer. See the manufacturer page for validation data and references: https://www.biolegend.com/en-us/products/brilliant-violet-421-anti-mouse-human-ki-67-antibody-13639
R&D 139NK050 R&D Systems™ Mouse NKG2D/CD314 Fc Chimera Recombinant Protein Activity is measured by its binding ability in a functional ELISA. Immobilized rmNKG2D/Fc Chimera at 4 µg/mL (100 µL/well) can bind biotinylated rmRae-1 gamma /Fc Chimera with a linear range of 1.56-100 ng/mL. See manufacturer's website for validation data: https://www.rndsystems.com/products/recombinant-mouse-nkg2d-fc-chimera-protein-cf_139-nk
Sinobiological 10575-H01S-50 Human NKG2D / CD314 / KLRK1 Protein (Fc Tag) HPLC-verified. See manufacturer's website for validation data: https://www.sinobiological.com/recombinant-proteins/human-nkg2d-10575-h01s
Fisher 50-112-8857 Invitrogen™ eBioscience™ Foxp3 / Transcription Factor Staining Buffer Set Buffer test is formulated and optimized for staining with antibodies against transcription factors and nuclear proteins (e.g. Foxp3 and Ki-67), cytokines and chemokines https://www.fishersci.com/shop/products/foxp3-transcription-factor-staining-buffer-set/501128857?searchHijack=true&searchTerm=50-112-8857&searchType=RAPID&matchedCatNo=50-112-8857
In vivo Antibodies
BioXcell BE0036 InVivoMAb anti-mouse NK1.1 Antibody has reported activity for in vivo NK depletion. NK cell depletion was confirmed in house with flow cytometry. See manufacturer's website for references: https://bioxcell.com/invivomab-anti-mouse-nk1-1-be0036#tab_pdetails
BioXcell BE0085 InVivoMAb mouse IgG2a isotype control, unknown specificity See manufacturer's wesbite for more detailed information. https://bioxcell.com/invivomab-mouse-igg2a-isotype-control-unknown-specificity-be0085#tab_specifications
BioXcell BE0090 InVivoMAb rat IgG2b isotype control See manufacturer's wesbite for more detailed information. https://bioxcell.com/invivomab-rat-igg2b-isotype-control-anti-keyhole-limpet-hemocyanin-be0090
BioXcell BE0003 In vivo MAb Anti-mouse CD4 Antibody has reported activity for in vivo CD4 depletion, flow cytometry and western blotting. CD4 depletion was confirmed in house by flow cytometry. See manufacturer's website for references: https://bioxcell.com/invivomab-anti-mouse-cd4-be0003-1
BioXcell BE0117 InVivoMAb anti-mouse CD8α Antibody has reported activity for in vivo CD8 depletion and western blotting. CD8 depletion was confirmed in house by flow cytometry. See manufacturer's website for references: https://bioxcell.com/invivomab-anti-mouse-cd8a-be0117
IHC and Western Blot
Abcam ab211327 Recombinant Anti-MASH1/Achaete-scute homolog 1 antibody Antibody is tested for IHC and WB by the manufacturer. See manufacturer's website for testing data: https://www.abcam.com/products/primary-antibodies/mash1achaete-scute-homolog-1-antibody-epr19840-ab211327.html
cell signaling technology #9662 Caspase-3 Antibody Antibody is tested for IHC and WB by the manufacturer. See manufacturer's website for testing data: https://www.cellsignal.com/products/primary-antibodies/caspase-3-antibody/9662
cell signaling technology 2203T TCF1/TCF7 (C63D9) Rabbit mAb Antibody is tested for IHC and WB by the manufacturer. See manufacturer's website for testing data: https://www.cellsignal.com/products/primary-antibodies/tcf1-tcf7-c63d9-rabbit-mab/2203
Cell Signaling 9718S Phospho-Histone H2A.X (Ser139) (20E3) Rabbit mAb Antibody is tested for IHC and WB by the manufacturer. See manufacturer's website for testing data: https://www.cellsignal.com/products/primary-antibodies/phospho-histone-h2a-x-ser139-20e3-rabbit-mab/9718
Cell Signaling 5483S Phospho-TBK1/NAK (Ser172) (D52C2) XP® Rabbit mAb Antibody is tested for IHC and WB by the manufacturer. See manufacturer's website for testing data: https://www.cellsignal.com/products/primary-antibodies/phospho-tbk1-nak-ser172-d52c2-xp-rabbit-mab/5483
Cell Signaling 3504S TBK1/NAK (D1B4) Rabbit mAb Antibody is tested for IHC and WB by the manufacturer. See manufacturer's website for testing data: https://www.cellsignal.com/products/primary-antibodies/tbk1-nak-d1b4-rabbit-mab/3504
Cell Signaling Technology 4302 IRF-3 (D83B9) Rabbit mAb Antibody is tested for IHC and WB by the manufacturer. See manufacturer's website for testing data: https://www.cellsignal.com/products/primary-antibodies/irf-3-d83b9-rabbit-mab/4302
Cell Signaling Technology 4947 Phospho-IRF-3 (Ser396) (4D4G) Rabbit mAb Antibody is tested for IHC and WB by the manufacturer. See manufacturer's website for testing data: https://www.cellsignal.com/products/primary-antibodies/phospho-irf-3-ser396-4d4g-rabbit-mab/4947
Fisher NC1651968 Cell Signaling Technology PHOSPHO-STING (SER365) (D8F4W) Antibody is tested for IHC and WB by the manufacturer. See manufacturer's website for testing data: https://www.cellsignal.com/products/primary-antibodies/tbk1-nak-d1b4-rabbit-mab/3504
Fisher NC0665193 STING (D2P2F) Rabbit mAb Antibody is tested for IHC and WB by the manufacturer. See manufacturer's website for testing data: https://www.cellsignal.com/products/primary-antibodies/sting-d2p2f-rabbit-mab/13647
Cell Signaling 13901S Vinculin (E1E9V) XP® Rabbit mAb #13901 Antibody is tested for IHC and WB by the manufacturer. See

manufacturer's website for testing data: https://www.cellsignal.com/products/primary-antibodies/vinculin-e1e9v-xp-rabbit-mab/13901
abcam ab5690 Anti-CD3 antibody Antibody is tested for IHC and WB by the manufacturer. See manufacturer's website for testing data: https://www.abcam.com/products/primary-antibodies/alpha-smooth-muscle-actin-antibody-ab5694.html
Fisher MA5-14520 Ki-67 Recombinant Rabbit Monoclonal Antibody (SP6)   Antibody is tested for IHC and WB by the manufacturer. See manufacturer's website for testing data: https://www.thermofisher.com/antibody/product/Ki-67-Antibody-clone-SP6-Recombinant-Monoclonal/MA5-14520
TIF Assay-Chrosome Spreads
PNA Bio F3002 Cy3 conjugated CENPB (ATTCGTTGGAAACGGGA) See the reference publications for the probe on manufacturer's website: https://www.pnabio.com/products/PNA_FISH.htm
Panagene F1002 Cy3-conjugated PNA Tel-C (CCCTAA)3 probe Not provided for this product http://www.panagene.com/_ENG/html/dh_product/prod_view/32/?cate_no=3
PNA Bio F1008 Alexa 488-conjugated PNA Tel-G (TTAGGG) See the reference publications for the probe on manufacturer's website: https://www.pnabio.com/products/PNA_FISH.htm
Millipore 5636 Anti-phospho-Histone H2A.X (Ser139) Antibody, clone JBW301 This Anti-phospho Histone H2A.X (Ser139) Antibody, clone JBW301, is validated for use in ICC for the detection of phospho Histone H2A.X (Ser139). See manufacturer's website for validation data. https://www.emdmillipore.com/US/en/product/Anti-phospho-Histone-H2A.X-Ser139-Antibody-clone-JBW301-Alexa-Fluor-647,MM_NF-05-636-AF647
Santa Cruz 376248 Lamin A/C Antibody (E-1): sc-376248 Lamin A/C Antibody (E-1) is a mouse monoclonal IgG1 κ Lamin A/C antibody is cited in 229 publications. See manufacturer's website for reference publications. https://www.scbt.com/p/lamin-a-c-antibody-e-1?requestFrom=search

Eukaryotic cell lines

Policy information about cell lines and Sex and Gender in Research

Cell line source(s)	Human SCLC lines (H69, H510, H2081, H841, H1048) were obtained from the Hamon Center for Therapeutic Oncology Research (UTSW). Mouse lines 984 and 984A were from Dr David McFadden (UTSW) and mouse RPP lines were from Dr Jane Johnson (UTSW).
Authentication	All human lines are routinely fingerprinted and mouse lines are genotyped routinely.
Mycoplasma contamination	All cell lines are mycoplasma negative.
Commonly misidentified lines (See ICLAC register)	we did not use any misidentified cell lines

Animals and other research organisms

Policy information about studies involving animals; ARRIVE guidelines recommended for reporting animal research, and Sex and Gender in Research

Laboratory animals	Mus musculus; B6129SF1/J; NU/J;NSG-SGM3;NSG-HLA-A2/HHD; 4-8 week-old for xenografts and syngeneic tumors.<br>4 week-old NSG-SGM3;NSG-HLA-A2/HHD for human immune system reconstitution for xenograft experiments.<br>16 week old of F1 hybrid of NSG-SGM3 and NSG-HLA-A2/HHD for xenografts.
Wild animals	The study did not involve wild animals.
Reporting on sex	Both sex was included in the study.
Field-collected samples	The study did not involve samples collected from the field.
Ethics oversight	All animal experiments described in this manuscript were approved by the<br>University of Texas Southwestern Institutional Animal Care and Use Committee.

Note that full information on the approval of the study protocol must also be provided in the manuscript.

Plants

Seed stocks	no plants were used in this study.
Novel plant genotypes	N/A
Authentication	N/A

Flow Cytometry

Plots

Confirm that:

- [x] The axis labels state the marker and fluorochrome used (e.g. CD4-FITC).
- [x] The axis scales are clearly visible. Include numbers along axes only for bottom left plot of group (a 'group' is an analysis of identical markers).
- [x] All plots are contour plots with outliers or pseudocolor plots.
- [x] A numerical value for number of cells or percentage (with statistics) is provided.

Methodology

Sample preparation	Cell Lines: Cell lines were stained with a fixable live/dead cell stain (Fisher Cat# 50-112-1528) for 8 minutes at room temperature, followed by staining with fluorophore-conjugated antibodies for 20 minutes on ice and in the dark. Cells were washed twice with a FACS buffer (2% FBS in PBS) after every staining step and analyzed using a BD FACS Canto machine or Cytek Northern Lights. Compensation was completed if samples were stained with multiple antibodies. The data were analyzed using the FlowJo software (v10.7).<br>Mouse tissues: Mouse tissues were minced and treated with DNase and collagenase (100 units/ml Collagenase IV Fisher Cat# 17104019, 10µg/ml DNase I Sigma Cat# DN25, 10% heat inactivated FBS in RPMI) for 1 hour at 37 degrees to dissociate the cells and then passed through a cell strainer (70µm) to create a single-cell suspension. Red blood cells (RBC) were removed from the sample using RBC lysis buffer (Fisher Cat# A1049201). These cells were then stained with a live/dead cell stain, followed by staining with fluorophore-conjugated antibodies as detailed above, following FC blocking with CD16/32 antibody (Biolegend, Cat# 101320) for 20 min on ice. The samples were washed twice after every step. To analyze intracellular markers, the samples were stained with surface markers and then permeabilized at 4 degrees overnight and incubated with fluorophore-conjugated antibodies recognizing intracellular markers. The manufacturer's instructions were followed for the intracellular staining (eBioscience Foxp3/Transcription Factor Staining Buffer Set, Thermofisher, Cat# 00-5523-00).<br>Flow cytometry analysis was performed using BDFACS Canto, and flow data was analyzed using FlowJo (v10.7). Compensation was performed for multi-color stains.
Instrument	Flow cytometry analysis was performed using BDFACS Canto, and flow data was analyzed using FlowJo (v10.7). Compensation was performed for samples containing multiple antibodies. Gating was performed to exclude dead cells from the analysis.
Software	BD FACSDiva v9.0 to collect flow cytometry data. FlowJo (v10.7) for analyzing flow cytometry data.
Cell population abundance	In mouse livers, 50%-75% among live cells after processing is CD45+ cells. 20%-30% of them is T cells. B cells make up 10% while NK cells make up 10%.
Gating strategy	Boundary between positive and negative are defined by two physically separate populations or corresponding isotype controls. Gating strategy for sorted cells and strategy for flow figures are shown as supplementary figures.

- [x] Tick this box to confirm that a figure exemplifying the gating strategy is provided in the Supplementary Information.
